# CRISPR/Cas9-Mediated Efficient Targeted Mutagenesis in Sesame (*Sesamum indicum* L.)

**DOI:** 10.3389/fpls.2022.935825

**Published:** 2022-07-11

**Authors:** Jun You, Donghua Li, Li Yang, Senouwa Segla Koffi Dossou, Rong Zhou, Yanxin Zhang, Linhai Wang

**Affiliations:** Key Laboratory of Biology and Genetic Improvement of Oil Crops, Ministry of Agriculture and Rural Affairs, Oil Crops Research Institute, Chinese Academy of Agricultural Sciences, Wuhan, China

**Keywords:** sesame, gene editing, CRISPR/Cas9, hairy root transformation, lignan

## Abstract

The clustered regularly interspaced short palindromic repeats (CRISPR)/CRISPR-associated protein 9 (Cas9) system has been widely utilized for targeted genome modification in a wide range of species. It is a powerful genome editing technology, providing significant benefits for gene functional research and molecular breeding. However, to date, no study has applied this genome editing tool to sesame (*Sesamum indicum* L.), one of the most ancient and important oil crops used widely in diverse industries such as food and medicine. Herein, the CRISPR/Cas9 system along with hairy root transformation was used to induce targeted mutagenesis in sesame. Two single guide RNAs (sgRNAs) were designed to target two sesame cytochrome P450 genes (*CYP81Q1* and *CYP92B14*), which are the key biosynthetic gene of sesamin and sesamolin, respectively. Sequencing data illustrated the expected InDel mutations at the target sites, with 90.63 and 93.33% mutation frequency in *CYP81Q1* and *CYP92B14*, respectively. The most common editing event was single nucleotide deletion and insertion. Sequencing of potential off-target sites of *CYP92B14*-sgRNA showed no off-target events in cases of three mismatches. High-performance liquid chromatography analysis showed that sesamin and sesamolin biosynthesis was effectively disrupted in the mutated hairy roots, confirming the crucial role of *CYP81Q1* and *CYP92B14* in sesame lignan biosynthesis. These results demonstrated that targeted mutagenesis was efficiently created by the CRISPR/Cas9 system, and CRISPR/Cas9 coupled with hairy root transformation is an effective tool for assessing gene functions in sesame.

## Introduction

Genome editing techniques offer scientists with opportunities to generate targeted DNA modification in plants leading to great advances in plant functional genomics as well as crop genetic improvement (Bao et al., 2019; Chen et al., 2019). Three targeted genome editing tools, that are zinc-finger nucleases (ZFNs), transcription activator-like endonucleases (TALENs), along with clustered regularly interspaced short palindromic repeats (CRISPR)/CRISPR-associated protein (Cas) system, have been well-developed for plant genome editing (Chen et al., 2019). These tools generate targeted DNA double-strand breaks (DSBs) and then achieve genome modifications *via* error-prone DNA repair medicated by either the non-homologous end-joining (NHEJ) cascade or the homology-directed repair (HDR) cascade (Hua et al., 2019). Among these genome editing tools, the RNA-guided CRISPR/Cas9 system has emerged as a simple, versatile, and robust tool for genome editing and has been widely employed to generate heritable genome modifications in a variety of plant species (Mao et al., 2019; Vats et al., 2019; Gao, 2021). In oil crops, such as oilseed rape, soybean, and cotton, CRISPR/Cas9-based genome-editing techniques have been successfully adopted to unveil the function(s) of genes associated with yields, plant architecture, quality, abiotic and biotic stress tolerance, and generate genome-edited crops with improved oil content, as well as fatty acid composition (Wu et al., 2020; Zhai et al., 2020; Zhang et al., 2020; Zheng et al., 2020; Chen et al., 2021). However, the efficiency and application of this system have not been reported in sesame.

Sesame (*Sesamum indicum* L.) is an ancient oilseed crop from the Pedaliaceae family, which has been cultivated in Asia for more than 5,000 years (Bedigian, 2004; Zhou et al., 2020). Sesame is used extensively for the production of edible oil, paste, and flour, and for improving the quality of diverse products because of its high oil contents, unique taste, and flavor (Hama, 2016). Sesame oil is regarded as quality edible vegetable oil due to its high content of unsaturated fatty acids (~80%) and possesses several beneficial bioactive components, including tocopherols, phytosterols, and lignans (Pathak et al., 2014).

Since the completion of the sesame genome sequencing, enormous genomic resources consisting of molecular markers, high-quality genetic maps, and various transcriptomic data are generated and used for sesame improvement (Dossa et al., 2017). However, the lack of a high-frequency genetic transformation system of sesame, severely restricts basic scientific research, especially gene functional studies in sesame. Nonetheless, *Agrobacterium rhizogenes*-mediated hairy root transformation has provided an alternative method for assessing gene function in non-model plants recalcitrant to genetic transformation. Hairy roots culture has been established in many plant species, and is widely utilized for phytochemical and recombinant protein production, rhizosphere physiology and biochemistry analysis, phytoremediation, and biosynthetic pathway elucidation (Ono and Tian, 2011; Georgiev et al., 2012). Several studies have demonstrated that combining hairy roots culture with the CRISPR/Cas9 technology is a rapid and efficient approach for functional genomics. For instance, Ron et al. (2014) have proved that *SHORT-ROOT* (*SHR*) gene's function is conserved between *Arabidopsis* and tomato through its knock-out using CRISPR/Cas9-mediated editing in transformed hairy roots. Shu et al. (2020) have revealed the function of the peanut *AhNFR5* in nodule formation *via* CRISPR-Cas9 targeting *AhNFRs* in transgenic hairy roots. CRISPR/Cas9-mediated gene targeting of *Cinnamoyl-CoA Reductase1* (*CCR1*) in *Eucalyptus* hairy roots confirmed the pivotal role of CCR1 in lignin biosynthesis as *CCR1*-edited lines have exhibited collapsed vessels and decreased lignification (Dai et al., 2020). In *Brassica carinata*, the knockout of *FASCICLIN-LIKE ARABINOGALACTAN PROTEIN 1 (BcFLA1)* by CRISPR-Cas9 mediated mutagenesis has resulted in a decrease of transgenic hairy roots' root hair under Pi (inorganic phosphate)-deficient conditions (Kirchner et al., 2018). Knocking out of *OpG10H* or *OpSLS* in *Ophiorrhiza pumila via* CRISPR/Cas9 have resulted in a decrease in camptothecin content, indicating that both *OpG10H* and *OpSLS* play critical roles in camptothecin biosynthesis (Shi et al., 2020). Sesame hairy roots culture has been developed for specialized metabolite production early in the 1990s (Ogasawara et al., 1993). However, hairy roots have not been employed as a platform for functional genomics research in sesame.

**Figure 1:**
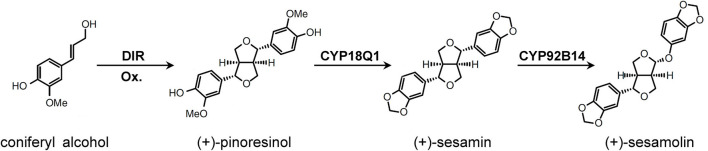
The lignans biosynthetic pathway in *Sesamum indicum*. Figure was drawn based on Ono et al. (2006) and Murata et al. (2017). DIR, dirigent protein; Ox, oxidant.

Sesamin and sesamolin are the primary lignans in sesame and have been found to exhibit antioxidant, antiaging, antihypertensive, anti-inflammatory, anticancer, and immunoregulatory activities (Majdalawieh and Mansour, 2019; Wu et al., 2019). Two cytochrome P450 proteins, CYP81Q1 and CYP92B14, have been demonstrated as the key enzymes that participated in lignan biosynthesis in sesame (Ono et al., 2006; Murata et al., 2017) (Figure 1). Herein, we tested the efficiency of the CRISPR/Cas9 system to create targeted genetic mutations in sesame using *CYP81Q1* and *CYP92B14* as targets. Guide RNAs targeting *CYP81Q1* and *CYP92B14* were designed by CHOPCHOP (Labun et al., 2019) and knockout mutants were formed *via Agrobacterium rhizogenes*-based hairy root transformation. The content of sesamin and sesamolin was considerably affected in *CYP81Q1*- and *CYP92B14*-mutated hairy roots. Our results demonstrated the applicability of the CRISPR/Cas9 system to induce specific mutations in sesame genes. Moreover, they confirmed the role of *CYP81Q1* and *CYP92B14*, and the potential of coupling CRISPR/Cas9 with hairy root transformation for assessing gene functions in sesame.

## Materials and Methods

### sgRNA Design and sgRNA:Cas9 Expression Vector Construction

Target sites for each gene were selected using the sgRNA design web tool CHOPCHOP (version 3) (http://chopchop.cbu.uib.no/), which enables to predict the frameshift rate of each gRNA and evaluate the on-target efficiency along with genome-wide off-targets (Labun et al., 2019). The genome assembly file and gene annotation file of *Sesamum indicum* obtained from the NCBI database were first submitted to CHOPCHOP. Then, all possible sgRNAs on the genomic sequence of *CYP81Q1* (*LOC105177765*) and *CYP92B14* (*LOC105175169*) were analyzed by CHOPCHOP in CRISPR/Cas9 knockout mode using gene ID as a query. One sgRNA for each gene was selected and their detailed information was presented in Supplementary Table S1.

To test the efficiency of the CRISPR-Cas9 system in sesame, the pKSE401 vector containing the maize-codon optimized *Cas9* expression cassette was used in this work. For every targeted locus, a pair of DNA oligos was generated and annealed to generate a double-stranded insert that has compatible ends with the *Bsa*I-digested empty vector. These dimers were inserted subsequently upstream of the sgRNA scaffolds in pKSE401 by the Golden Gate assembly method using *Bsa*I and T4 Ligase (New England BioLabs) as described previously (Xing et al., 2014). All constructed vectors were confirmed *via* sequencing. The two binary vectors, *CYP81Q1-gR* and *CYP92B14-gR*, each harboring one gRNA, were introduced into the *Agrobacterium rhizogenes* strain K599 for sesame hairy roots transformation. Primers employed for the construction of the vector are given in Supplementary Table S2.

### Plant Materials

Sesame genotype G98 was utilized for hairy roots transformation. Seeds of G98 were surface sterilized by 10-min treatment in a 3% sodium hypochlorite solution and then placed on MS (Murashige and Skoog) agar medium at 28°C under 16 h light/8 h dark photoperiod for 7–10 days.

### Sesame Hairy Root Transformation

The cotyledons were cut from seedlings and used as explants for hairy roots' induction and transformation following the methods described by Chun et al. (2009) with some modifications. In brief, *Agrobacterium rhizogenes* K599 bearing either *CYP81Q1-gR* or *CYP92B14-gR* were streaked on solid LB medium harboring 50 mg/L kanamycin followed by incubation for 3 days at 28°C. Then, a single colony picked from the plate was grown overnight at 28°C in LB liquid medium harboring 50 mg/L kanamycin. Bacterial cells were collected by spinning at 5,000 rpm for 10 min and the pellet was re-suspended in MS liquid medium to an OD_600_ of about 0.6–0.8. Dissected cotyledons that were softly wounded with scalpel blades were dipped in the prepared bacterial suspension for 10 min. Cotyledons were then co-cultured on 1/10 MS agar medium harboring 20 mg/L acetosyringone for 3 days. After washing with MS liquid medium harboring 500 mg/L cefotaxime, cotyledons were blot-dried on sterile filter paper, then placed on half-strength MS agar medium harboring 100 mg/mL kanamycin and 200 mg/L timentin at 28°C under 16 h light/8 h dark photoperiod. After 2 weeks, the hairy roots derived from cotyledons were excised and cultured on fresh half-strength MS agar medium harboring kanamycin and timentin. The rapidly growing hairy roots selected on the MS agar medium were cultured in a shaking flask in darkness at 28°C.

### Identification of Transformed Hairy Roots

Transgenic hairy roots carrying T-DNA insertions were assessed *via* PCR using the Plant T5 Direct PCR Kit (Tsingke Biotechnology Co., Ltd.). The list of primers is provided in Supplementary Table S2. Gene-specific primers for *rolB* were designed based on the sequences of the *rolB* gene in *Agrobacterium rhizogenes* strain K599, plasmid pRi2659 (GenBank accession number: EF433766.1). Primers used for sgRNA expression cassette verification were reported in a previous study (Xing et al., 2014). Plasmid *CYP81Q1-gR* or *CYP92B14-gR* and wild-type normal roots were utilized as positive and negative controls, respectively. The PCR reaction conditions were as follows: pre-denaturation at 94°C for 5 min; 30 cycles at 94°C for 30 s, 55°C for 30 s, and 72°C for 30 s; and final extension at 72°C for 2 min. Finally, PCR products were analyzed by 1.0% agarose gel electrophoresis.

### Detection of On-Target Mutations in Cas9:sgRNA-Expressing Hairy Roots

For mutation analysis, the genomic region harboring target sites from positive hairy root lines were amplified by PCR using Plant T5 Direct PCR Kit (Tsingke Biotechnology Co., Ltd.). The PCR amplicons were sequenced using specific internal primers located in 100 to 200 bp upstream or downstream of the targeted sites. Sequencing chromatograms with superimposed spectra were directly analyzed by a highly reliable Degenerate Sequence Decoding (DSD) approach in the web-based tool DSDecode (http://skl.scau.edu.cn/dsdecode/) (Liu et al., 2015). The PCR products that contained complex mutations that could not be decoded in DSDecode were subcloned into pGEM-T vectors, and at least 10 clones from each amplicon were sequenced. Primers used for PCR amplification and sequencing were listed in Supplementary Table S2.

### Off-Target Analysis

In the process of designing sgRNAs using CHOPCHOP, predicted off-target sites of target sequences were identified. Two predicted off-target sites of CYP92B14 sgRNA were selected for off-target analysis. Specific primers were designed and used to amplify genomic DNA fragments harboring potential off-target sites. The sequence information of off-target sites was analyzed as described above.

### Evaluation of Sesamin and Sesamolin Contents

Hairy roots were collected and dried at 50°C for lignans analysis. Samples were extracted according to Wang et al. (2013) with some modifications. In brief, dried hairy roots samples were ground into powder. Each sample was weighed 0.2 g powder, and 5 mL anhydrous ethanol was added following extraction at 220 rpm for 2 h. Next, samples were spanned at 8,000 rpm for 5 min. The supernatant was lyophilized using a freeze dryer (ScientZ-30D) and the residue was dissolved in 1 mL of 80% ethanol by centrifugation at 200 rpm for 30 min. The content of sesamin and sesamolin was analyzed *via* high-performance liquid chromatography using Agilent 1260 Infinity II (Agilent Technologies, Waldbronn, Germany) according to the method described previously (Xu et al., 2021).

## Results

### Construction of sgRNA:Cas9 Expression Vector

**Figure 2:**
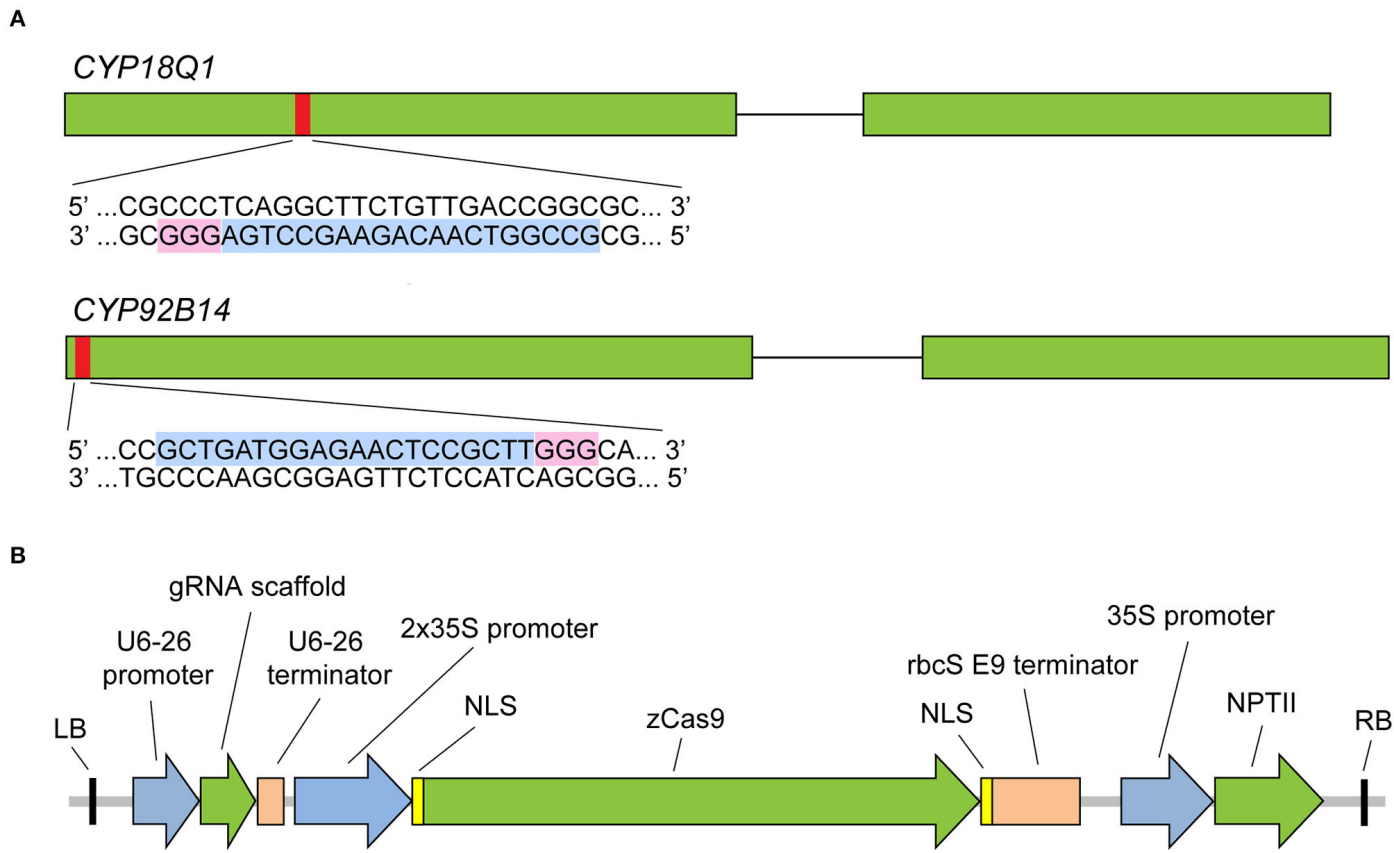
CRISPR/Cas9 sgRNA design and CRISPR/Cas9 binary vector. **(A)** Diagram illustrating *CYP81Q1* and *CYP92B14* gene structure and the selected target sequences. Green boxes denote exons, while black lines denote introns. The red boxes denote each gene target site, and sgRNA target sequence and PAM sequences are highlighted in light blue and pink, respectively. **(B)** The binary vector that was created for the CRISPR/Cas9 system is shown. RB and LB, right and left borders; *zCas9, Zea mays* codon-optimized *Cas9*; NLS, nuclear localization sequence; *NPTII*, neomycin phosphotransferase II gene.

To test the ability of sgRNA-mediated CRISPR/Cas9 system to effectively trigger a gene-specific modification in *Sesamum indicum*, two cytochrome P450 genes, *CYP81Q1* and *CYP92B14* (Ono et al., 2006; Murata et al., 2017), involved in lignan biosynthesis were selected as the targets for gene editing. Both genes have a single copy in the sesame genome and contain two exons. Specific sgRNAs were designed via a web resource CHOPCHOP (version 3) (http://chopchop.cbu.uib.no/) (Labun et al., 2019). A 20-bp sequence with NGG protospacer adjacent motif (PAM) in their 3′-regions of the first exon of each gene was selected for targeting by sgRNA (Figure 2A, Supplementary Table S1). In this study, the CRISPR/Cas9 system that has been successfully applied in *Arabidopsis* and maize (Xing et al., 2014) was tested. As illustrated in Figure 2B, the binary vector harbors maize-codon optimized *Cas9* expression cassette under dual cauliflower mosaic virus (CaMV) 35S promoter and the neomycin phosphotransferase (NPTII) selection marker gene. Two separate binary vectors (*CYP81Q1-gR* and *CYP92B14-gR*), each containing one sgRNA driven by *Arabidopsis* U6-26 promoter, were constructed.

### Screening Along With the Characterization of Transgenic Hairy Roots

**Figure 3:**
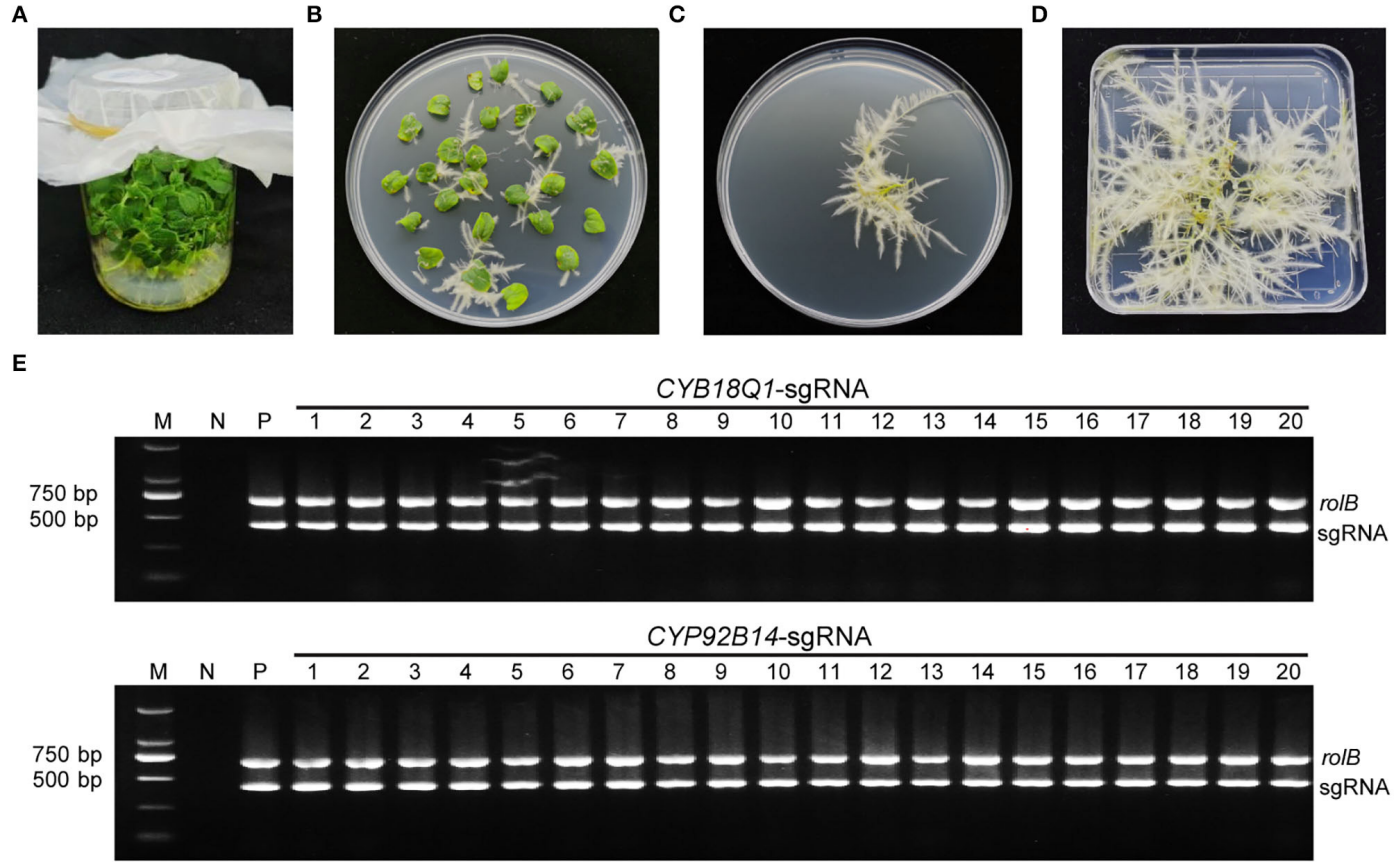
Development and characterization of positive transgenic hairy roots. **(A)** Aseptic sesame seedlings cultivated on MS medium. **(B)** Transgenic hairy roots from cotyledon explants harboring the CRISPR/Cas9 vector. **(C)** Kanamycin-selected positive transgenic hairy roots. **(D)** Positive hairy roots were cultured for 2 weeks. **(E)** Characterization of positive transgenic hairy root lines. Each hairy root line genomic DNA was isolated and utilized as a template for PCR employing particular primers for the *rolB* genes along with the sgRNA expression cassette. The *CYP81Q1-gR* and *CYP92B14-gR* plasmids were utilized as a positive control (P), and genomic DNA from wild-type normal roots was utilized as negative control (N).

*Agrobacterium rhizogenes* strain K599 harboring *CYP81Q1-gR, CYP92B14-gR*, or empty vector were used for the hairy root transformation. After 10 days of infection, hairy roots emerged from wounded cotyledons (Figures 3A–D). Positive transgenic roots carrying CRISPR/Cas9 binary vector were verified by PCR assays using specific primers for the *rolB* genes and sgRNA expression cassette (Supplementary Table S2). The expected 701 bp (for *rolB*) and 423 bp (for sgRNA) amplified product were observed in positive transgenic hairy root lines (Figure 3E).

### Analysis of CRISPR/Cas9-Mediated Mutagenesis

To investigate the efficiency of the CRISPR/Cas9 system for inducing mutagenesis in sesame and characterize the mutation types, target gene regions spanning sgRNA sequence were PCR amplified from positive transgenic hairy roots and then subjected to Sanger sequencing. A total of 32 and 30 independent transgenic lines in which *CYP81Q1* and *CYP92B14* were targeted, respectively, were used and analyzed. Sequencing chromatograms from each line were manually checked, and mutations were concluded successfully introduced when nucleotide changes (deletion, insertion, or substitution) or multiple overlapping traces were shown at the sgRNA target sites (Supplementary Figure S1). The results indicated a high mutagenesis frequency of 90.63% (29 out of 32) and 93.33% (28 out of 30) occurred in *CYP81Q1* and *CYP92B14* transgenes, respectively (Table 1). Sixteen homologous mutations were detected for two targets, representing 25.81% of all transgenic hairy root lines or 28.07% in hairy roots harboring targeted mutations.

**Table 1:** Summary of mutations in transgenic hairy roots.

**Gene**	**Total transgenic**	**Edited**	**% Mutation**	**Homozygous**	**Biallelic**	**Monoallelic**	**Chimera**
	**lines**	**lines**	**frequency**	**mutant lines**	**mutant lines**	**mutant lines**	
*CYP81Q1*	32	29	90.63	6	20	2	1
*CYP92B14*	30	28	93.33	10	14	3	1

**Figure 4:**
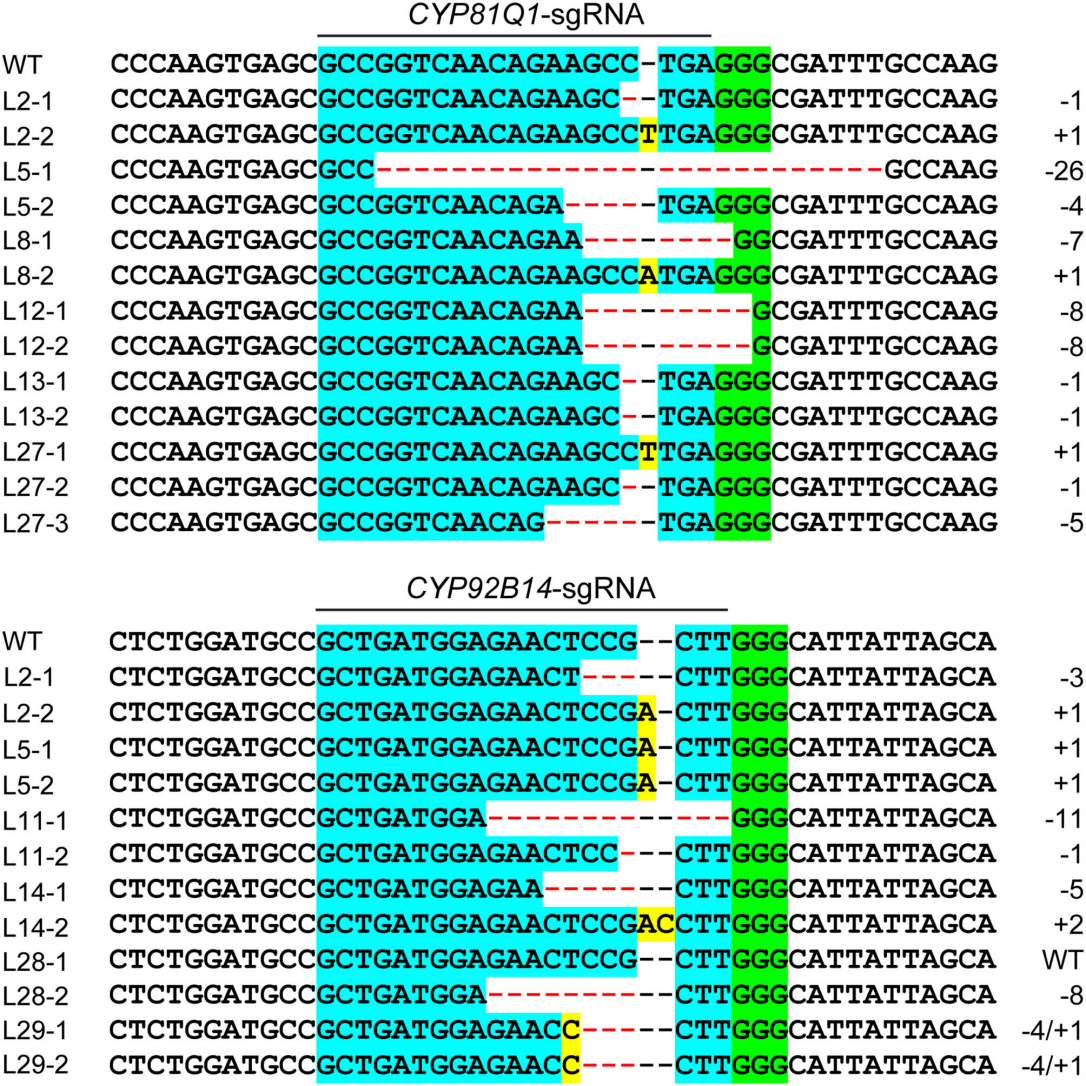
Sequence analysis of CRISPR/Cas9-triggered targeted mutations in *CYP81Q1* and *CYP92B14*. Representative sequence alignments of six transgenic hairy root lines from each gene were shown. The sgRNA targets are indicated with light blue background and the PAM motif (NGG) is indicated with green background. The insertion and deletion of nucleotides are indicated with yellow background and red dashes, respectively. Mutation type insertion (+), deletion (–), and size are provided on the panel's right side.

**Figure 5:**
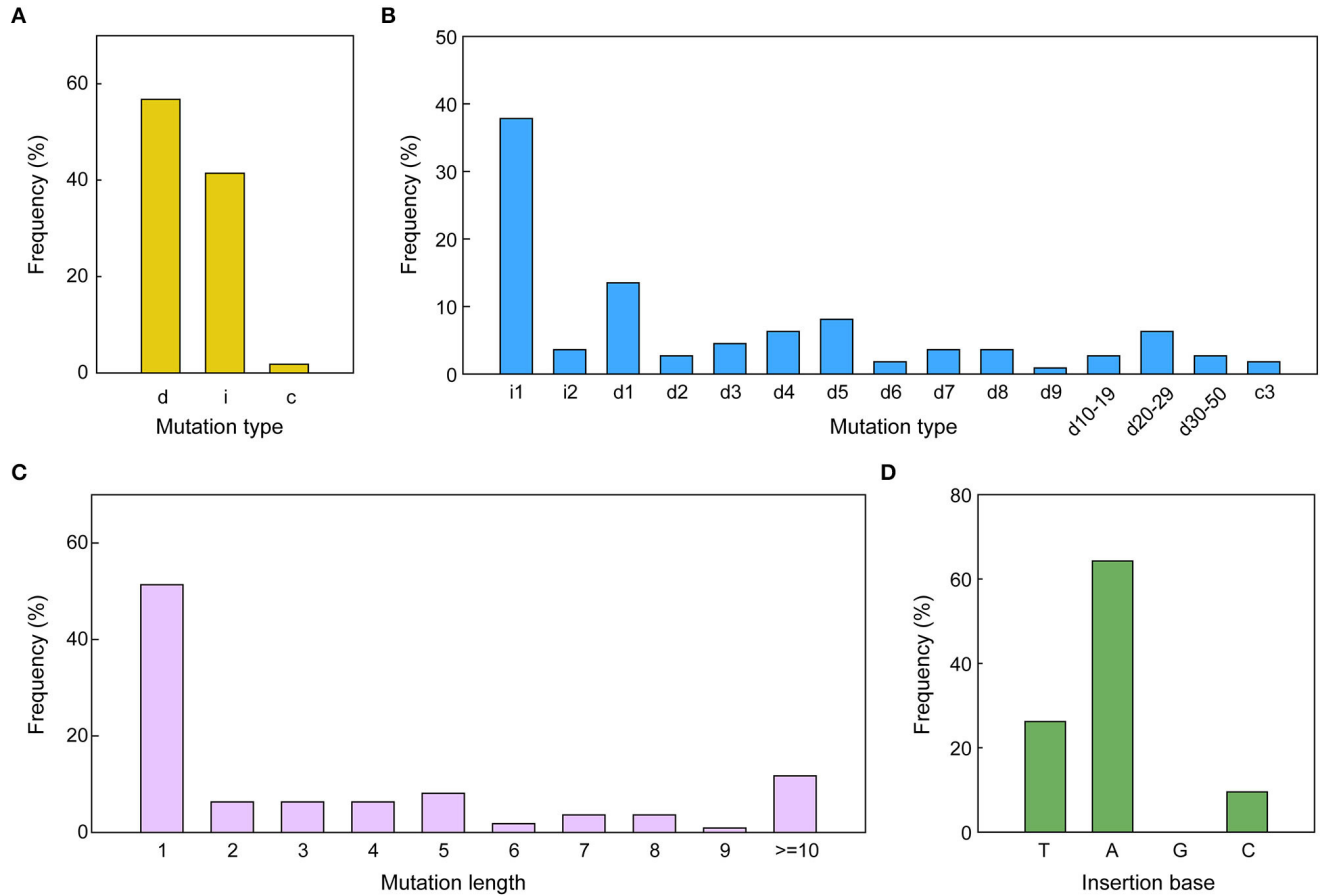
CRISPR/Cas9-triggered mutation types along with frequency in sesame. **(A,B)** Mutation kinds along with the frequency of all mutations induced by the two different sgRNAs. i, insertion; c, combined mutation; d, deletion. i#, number of bp inserted at the target site; d#, number of base pairs (bp) deleted from the target site; c#, number of bp combined mutations. **(C)** The frequency of various mutation lengths irrespective of the mutation type. **(D)** Percentage of bases inserted for the 1-bp insertions. A, adenine; C, cytosine; T, thymine; G, guanine.

The pattern and frequency of CRISPR/Cas9 triggered mutations in sesame were analyzed based on sequencing data of two target sites. The detailed mutations for every edited allele are provided in Supplementary Datasheets S3, S4. The results showed that three types of mutations occurred in the edited alleles: deletions, insertions, and combined mutations (more than one mutation type in one allele), with a frequency of 56.76, 41.44, and 1.80%, respectively (Figures 4, 5A). The length of mutations ranged from 1 to 46 bp, a majority of which (51.35%) were single-base mutations (Figure 5C). Only 11.71% of mutations exhibited a >10 bp deletion. A total of 23 edition types were detected in edited alleles of all target sites, with 37.84% of the mutations belonging to one nucleotide insertion (Figure 5B). Most inserted single base was Adenine (64.29%) or Thymine (26.19%), while single Guanine insertions were not detected in the examined transgenic lines (Figure 5D).

The mutation types of each target gene were further analyzed (Supplementary Figure S2). The *CYP81Q1*-sgRNA triggered deletions at a frequency of 78.95% (Table 2), mainly 1 bp deletions (24.56%) and 2–5 bp short deletions (24.56%). For *CYP92B14*, 1.85% and 18.52% of the alleles exhibited 1 bp deletions and 2–5 bp short deletions, respectively. Overall, 18 out of 54 alleles (33.33%) had deletions caused by *CYP92B14*-sgRNA (Table 2). In *CYP81Q1* edited lines, 10 alleles had 1 bp insertion and no 2 bp insertions were observed. Whereas, in *CYP92B14* lines, 30 and 4 alleles showed 1 and 2 bp insertions, respectively, representing 62.96% (34 alleles out of 54 alleles) of the editing events. It is worth noting that the majority of edited alleles of *CYP81Q1* (91.23%) and *CYP92B14* (88.89%) led to reading frame shifts or large deletions (≥30 bp) without affecting the reading frame (Supplementary Table S3), indicating that the activity of the corresponding protein was potentially altered.

**Table 2:** Characterization of mutation types in each target gene.

**Gene**	**Total edited**	**Total edition**	**Deletion**				**Insertion**		**Combined**
	**alleles**	**types**							**mutation**
			**1 bp**	**2–5 bp**	**6–10 bp**	**>10 bp**	**1 bp**	**2 bp**	
*CYP81Q1*	57	18	14 (24.56%)	14 (24.56%)	7 (12.28%)	10 (17.54%)	12 (21.05%)	0	0
			45 (78.95%)				12 (21.05%)		
*CYP92B14*	54	13	1 (1.85%)	10 (18.52%)	4 (7.41%)	3 (5.56%)	30 (55.56%)	4 (7.41%)	2 (3.70%)
			18 (33.33%)				34 (62.96%)		

Sesame is a diploid species with two alleles per gene. Thus, one or both alleles of the target genes might be cleaved and mutated by the CRISPR/Cas9 system. For *CYP81Q1*-edited lines, 20 out of 29 (68.97%) were biallelic mutations with two different mutated alleles (Table 1). Six lines (20.69%) exhibited homozygous mutation with two identical mutated alleles. Only two accounting for 6.90% were monoallelic mutant lines with a mutated allele along with a WT allele. Out of 28 *CYP92B14*-edited lines obtained, 14 (50%) were biallelic mutations, 10 (35.71%) were homozygous, 3 (10.71%) were monoallelic mutants, and 1 (3.57%) was a chimera (Table 1). Although the transgenic lines had similar editing efficiency, the genotype distribution of the mutated hairy roots of the two genes was quite different. Biallelic mutations were the main type for *CYP81Q1*-edited lines, while *CYP92B14*-edited lines had more homozygous mutations. Of all the transgenic hairy roots examined, 25.81% (16 out of 62) were homozygous and 54.84% (34 out of 62) were biallelic mutations, giving a total of 80.65%. The predominant editing type of homozygotes was 1 bp insertions (50%), followed by 1 bp deletions (12.5%) (Supplementary Figure S3A). For the biallelic mutations, no dominant type was detected (Supplementary Figure S3B). *In silico* analysis revealed that 69.35% (43 out of 62) of the transgenic hairy root lines had InDel mutations causing significant protein modifications (reading frame shifts or more than 10 aa deletion) in both alleles, inferring potential knockout of the target genes.

### Off-Target Analysis in the Transgenic Hairy Roots

The putative off-target sites of each targeted gene were assessed with the online tool CHOPCHOP. No putative off-target site was detected in *CYP81Q1-*sgRNA. Two putative off-target sites were identified in *CYP92B14*-sgRNA (Table 3), with one and three mismatches, respectively. To probe for off-target events in *CYP92B14* edited transgenic hairy roots, we cloned DNA fragments harboring the putative off-target sites *via* PCR with specific primers (Supplementary Table S2). Sequencing data of PCR amplicons showed that no mutation occurred at the putative off-target sites with three mismatches in the tested transgenic hairy roots (Supplementary Figure S5). In contrast, we found that mutations occurred at putative off-target sites with one mismatch in 86.67% (13 out of 15) tested transgenic hairy roots (Table 3, Supplementary Figure S4).

**Table 3:** Analysis of mutation in potential off-target sites.

**Target**	**Off-target**	**Putative off-**	**Putative off-**	**Number of**	**Number of lines**
	**sites**	**target sequences**	**target loci**	**examined lines**	**lines with off-targets**
CYP92B14-sgRNA	1	GCTGATGGAGAACTCCTCTT*GGG*	NC_026146.1:331676	15	13
	2	CCATATCGGTGTTCTCCATC*AGC*	NC_026146.1:9970338	15	0

*PAM sequence (NGG and the analog NGC) is indicated in italic. Mismatched nucleotides are marked with an underline*.

### Lignan Content of Mutant Hairy Root Lines

**Figure 6:**
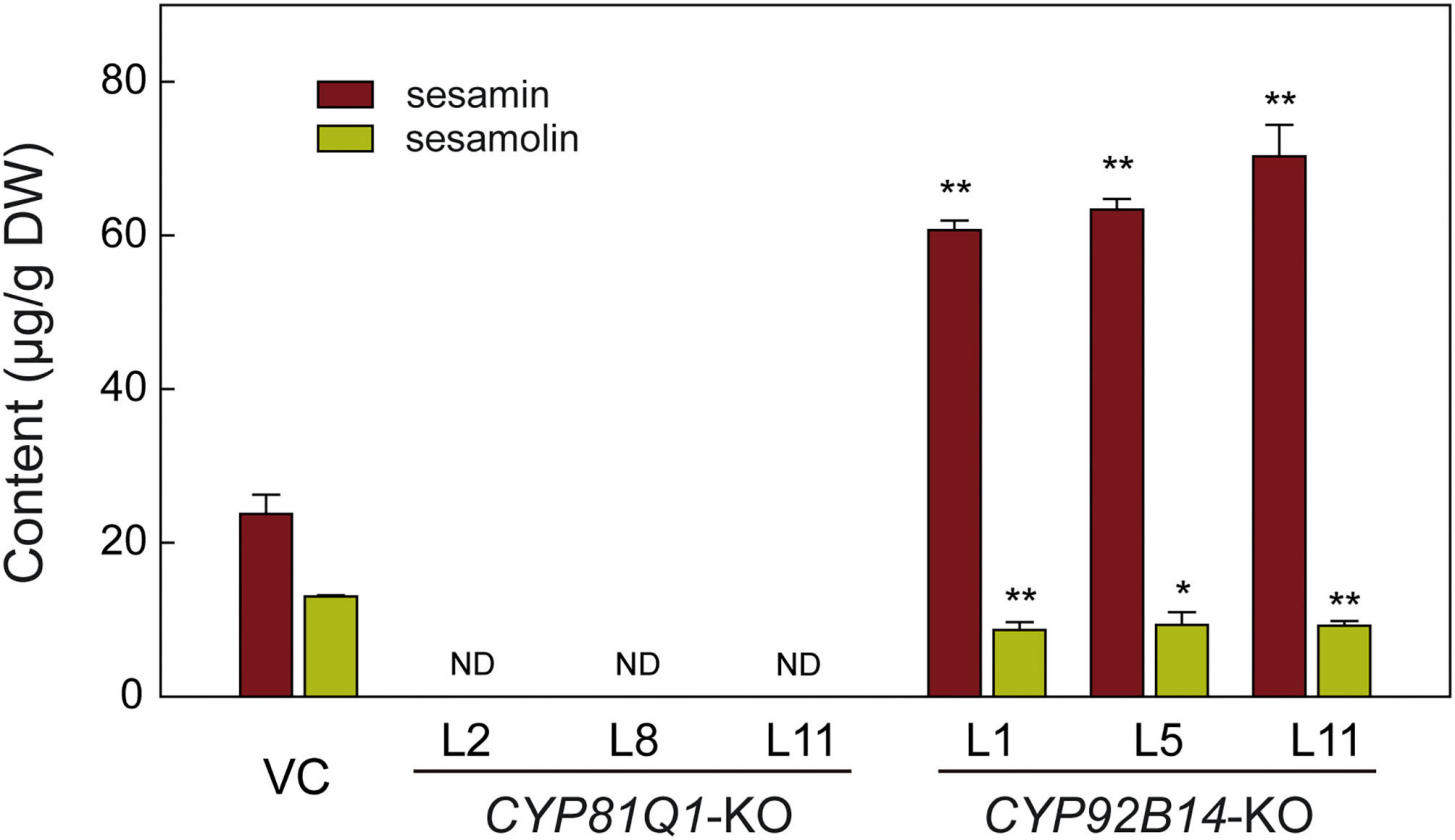
Lignans content analysis in *CYP81Q1*- and C*YP92B14*-knockout hairy roots. The contents of two lignans (sesamin and sesamolin) in vector control (VC) lines, and the *CYP81Q1*- and *CYP92B14*-knockout (KO) lines. DW, Dry weight; ND, Not detected. Error bars designate the standard error (*n* = 3), **P < 0.01; *P < 0.05, *t*-test.

To uncover the effects of CRISPR/Cas9-mediated mutation of *CYP81Q1* or *CYP92B14* on lignans biosynthesis and accumulation, three knockout lines (KO) of each gene were chosen for sesamin and sesamolin contents evaluation *via* HPLC (high-performance liquid chromatography). As shown in Figure 6, both sesamin and sesamolin were not detected in *CYP81Q1*-knockout lines, indicating a successful breakdown of the sesame-specific lignans pathway. In *CYP92B14*-knockout lines, the content of sesamolin was significantly (*t-*test, *P* < 0.05) decreased, while the sesamin content was significantly (*t-*test, *P* < 0.01) increased (Figure 6). The content of sesamin in *CYP92B14*-knockout lines was 1.72-fold higher than in vector control (VC) lines, denoting that the conversion of sesamin to sesamolin was altered yielding to sesamin accumulation. These results confirmed the essential role of *CYP81Q1* and *CYP92B14* in sesame lignan biosynthesis.

## Discussion

The CRISPR/Cas9 system has been extensively employed for targeted DNA alteration as a simple, extremely effective, and adaptable genome editing approach, and has demonstrated enormous promise in functional genomics research and molecular breeding (Bao et al., 2019; Gao, 2021). Herein, the CRISPR/Cas9 system combined with the hairy roots transformation system was adopted to trigger site-directed deletions along with insertions in the genome of the non-model plant sesame. This work expanded the application of the CRISPR/Cas9 system in the plant kingdom and provided a cost-effective and highly efficient platform for functional genomics in sesame.

Although CRISPR/Cas9 system appears to be generally effective, the efficiency of targeted mutagenesis varies depending upon the target sites, sgRNA sequences, promoters driving *Cas9* and sgRNAs, codon-optimization of *Cas9*, as well as transformation method (Ma et al., 2016). As previously reported, the mutagenesis effectiveness of 82.6% and 60% were detected in *Arabidopsis* and maize, respectively, by *Agrobacterium*-mediated transformation (Xing et al., 2014). While, PEG-medicated protoplast transformations of *Arabidopsis* and rice targeting different endogenous genes showed only 6.5% and 7.3% mutation efficiency, respectively (Lin et al., 2018). Herein, we investigated the efficiencies of the CRISPR/Cas9 system delivery of mutations in *CYP81Q1* and *CYP92B14*, two cytochrome P450 genes that catalyze the main sesame lignans, sesamin, and sesamolin synthesis, respectively. We obtained mutagenesis efficiency of 90.63 and 93.33% for *CYP81Q1* and *CYP92B14*, respectively, in transgenic sesame hairy roots. These efficiencies are similar to or higher than those achieved in hairy roots of soybean (10–93.3%) (Cai et al., 2015), peanut (50–80%) (Shu et al., 2020), *Salvia miltiorrhiza* (11.5–30.8%) (Li et al., 2017), and *Eucalyptus* (51.7–89.9%) (Dai et al., 2020). Several investigations have illustrated that expression of *Cas9* driven by promoters with elevated activity in the germline cell or dividing tissue leads to higher mutation frequency compared with a constitutive promoter in *Arabidopsis* (Wang et al., 2015; Yan et al., 2015). In monocotyledons, *Cas9* under the control of the constitutive *Ubiquitin* promoter has generated higher editing efficiency relative to the CaMV *35S* promoter (Ma et al., 2016). Investigations in soybean and grape have shown that endogenous U6 promoters that induce the expression of sgRNA could increase the genome editing efficiency in contrast with *Arabidopsis* U6 promoter (Sun et al., 2015; Ren et al., 2021). The high percentages (over 90%) of hairy root mutants generated in our study indicated that the *Arabidopsis* U6 and 2× CaMV 35S promoters modulating the expression of sgRNAs and the maize codon-optimized *Cas9*, respectively, could be efficiently utilized for genome editing and functional genomics studies in sesame hairy roots.

To accurately determine the genotype of the resultant mutants, sequencing of the mutated sites is required. Herein, the targeted mutations were determined by direct sequencing of amplicons followed by decoding into allelic sequences *via* the web-based tool DSDecode (http://skl.scau.edu.cn/dsdecode/) (Liu et al., 2015). Using this method, the allele sequence of the target site can be obtained quickly without laborious and tedious cloning operation and sequencing of multiple clones. Analysis of sequencing data revealed that the main types of mutation in sesame were single nucleotide deletion and insertion, consistent with reports in other plants (Zhang et al., 2014, 2019; Wang et al., 2018; Zhu et al., 2018; Wu et al., 2020; Zheng et al., 2020). Although a high percentage of edited alleles in both targets were found in the present study, mutation types and frequency were distinct among different target sites. The *CYP81Q1* mutations were primarily deletion, with a considerable proportion of mutations that involved 1 to 5 bps. In contrast, high frequencies of short (1–2 bp) insertions were found in *CYP92B14*. Similar differences across different target genes were also reported in rice (Zhang et al., 2014) and wheat (Zhang et al., 2019). These differences might be attributed to differences in the CRISPR/Cas9 system activity and/or the base composition between different targets (Zhang et al., 2014; Ma et al., 2015). Genotype analyses revealed that 80.65% of the sesame hairy roots were homozygous or biallelic for the target genes. The majority (86%) of these mutations, representing 69.35% of all transgenic hairy root lines, may render knockout of target genes due to the introduction of frameshifts InDel mutation and/or large fragment deletion in both alleles. Efficiently created homozygous and biallelic mutations by CRISPR/Cas9 to obtain loss-of-function mutants is important for gene functional characterization. Many studies applied two or more sgRNAs targeting different sites of signal genes to achieve a higher gene editing efficiency with large fragment deletions (Fan et al., 2015; Wang et al., 2018; Bernard et al., 2019; Zheng et al., 2020). In the present study, we found that a single sgRNA could efficiently generate a knockout mutant of the target gene in sesame hairy roots, which is ideal for further studies. Meanwhile, the use of a single sgRNA also may help reduce the risk of off-target mutations.

The targeting specificity of the CRISPR/Cas9 system is controlled by the guide RNA sequence and the PAM next to the target sequence. Several studies have shown the occurrence of off-target edition with mismatch number and position in the sgRNA sequence (Hsu et al., 2013; Xie and Yang, 2013). For example, sgRNA with 1 or 2 bp mismatch to the off-target site has a high off-target frequency (47.5–67.5%) in rice, while the off-target rate was only 2.5% for sgRNA with six mismatches (Li et al., 2016). Herein, a high off-targeting rate (86.67%) was found for *CYP92B14*-sgRNA with a single mismatched base between the sgRNA and its off-target site. Tolerance of the CRISPR/Cas9 system to single nucleotide mismatch observed in sesame is in accordance with other studies in rice and human cells (Fu et al., 2013; Zhang et al., 2014). For species that have had their genomes sequenced, the selection of highly specific targets through online tools (such as CHOPCHOP) is one of the effective strategies to avoid off-target mutations.

*Agrobacterium rhizogenes*-mediated hairy root culture has been utilized as a powerful tool for diverse secondary metabolites production and functional genomics research. For example, hairy root transformation is widely used for uncovering the specific functions of genes involved in legume-rhizobia symbiosis (Hernandez-Lopez et al., 2019; Zhang et al., 2021). Hairy root culture has also provided a valuable platform for producing valuable secondary metabolites and elucidating biosynthetic pathways in plants (Shi et al., 2021). Several transcription factors, including *IiWRKY34* and *Ii049*, were identified as the key genes controlling lignan biosynthesis in *Isatis indigotica* based on transgenic hairy roots (Ma et al., 2017; Xiao et al., 2020). Early in the 1990s, high yield of naphthoquinone was achieved through sesame hairy root culture (Ogasawara et al., 1993). Later, sesame hairy roots were used to produce recombinant fungal phytase and elucidate the biosynthetic pathway of naphthoquinone and anthraquinone derivatives (Chun et al., 2007; Furumoto and Hoshikuma, 2011; Furumoto and Sato, 2017). However, functional characterization of sesame genes using a hairy root transformation system has not been reported so far. In this study, two genes related to lignans synthesis (*CYP81Q1* and *CYP92B14*) were efficiently mutated in sesame hairy roots by CRISPR/Cas9-mediated gene editing. Consequently, the content of corresponding lignans in transgenic lines was significantly affected as expected. The content of sesamin in the *CYP92B14*-mutated transgenic lines was 2.7 times higher than that of control lines, confirming the possibility of sesamin production in sesame hairy roots through metabolic engineering. These results confirm the crucial role of these two genes in sesamin and sesamolin biosynthesis, respectively. More importantly, they demonstrate that the hairy root system is a promising alternative model system for effective dissection and validation of metabolism-related genes' functions in sesame. Furthermore, our findings may stimulate studies on the sesame lignans biosynthesis pathway and the underlying regulatory mechanisms for biotechnological applications.

## Conclusion

In this study, efficient genome editing was successfully achieved in sesame using the CRISPR/Cas9 system through hairy root transformation mediated by *A. rhizogenes* K599. Two sgRNAs targeting the sesamin (*CYP81Q1*) and sesamolin (*CYP92B14*) biosynthetic gene, respectively, triggered mutagenesis at the target sites with frequencies over 90%. HPLC analysis illustrated that the content of the two lignans was significantly affected in *CYP81Q1*- and *CYP92B14*-mutated hairy roots. The high proportion of homologous and biallelic mutants acquired in the present study implies that CRISPR/Cas9-mediated knockout is a promising and efficient strategy for loss-of-function studies in sesame. Moreover, CRISPR/Cas9 system coupled with hairy root transformation was approved as an effective alternative for deciphering gene functions and complex regulatory networks in sesame.

## Data Availability Statement

The original contributions presented in the study are included in the article/Supplementary Material, further inquiries can be directed to the corresponding author/s.

## Author Contributions

JY and LW: conceptualization. JY, DL, and LY: methodology, investigation, validation, and visualization. RZ: data curation. YZ: resources. JY: writing—original draft preparation. JY, SD, and LW: writing—review and editing. LW: supervision and funding acquisition. All authors contributed to the article and approved the submitted version.

## Funding

This research was funded by the Agricultural Science and Technology Innovation Project of the Chinese Academy of Agricultural Sciences (CAAS-ASTIP-2016-OCRI), the China Agriculture Research System (CARS-14), the Fundamental Research Funds for Central Non-profit Scientific Institution (Y2022XK11), the Key Research Projects of Hubei province (2020BBA045 and 2020BHB028), and the Science and Technology Innovation Project of Hubei province (201620000001048). The funders had no role in the experimental design, data collection and analysis, or writing the manuscript.

## Conflict of Interest

The authors declare that the research was conducted in the absence of any commercial or financial relationships that could be construed as a potential conflict of interest.

## Publisher's Note

All claims expressed in this article are solely those of the authors and do not necessarily represent those of their affiliated organizations, or those of the publisher, the editors and the reviewers. Any product that may be evaluated in this article, or claim that may be made by its manufacturer, is not guaranteed or endorsed by the publisher.
