# Supplementary material for: CRISPR/Cas9-Mediated Efficient Targeted Mutagenesis in Sesame (Sesamum indicum L.)
Source: Front Plant Sci. 2022 Jul 11;13:935825. doi: 10.3389/fpls.2022.935825 (PMC9309882; doi:10.3389/fpls.2022.935825)
Supplement: Supplementary file 1 [file Data_Sheet_1.pdf]

Supplementary Figures

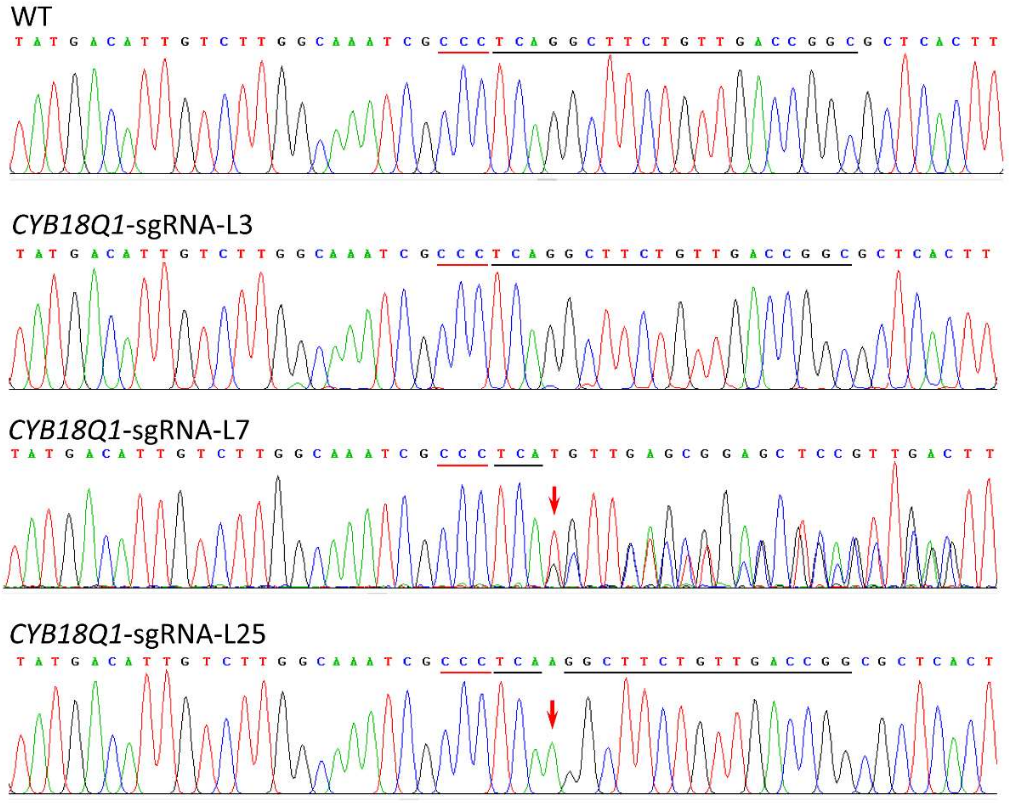

Figure S1. Examples of sequencing chromatograms from WT and transgenic hair roots. No mutation was detected in *CYB18Q1*-sgRNA-L3. The red arrow indicates overlapping peaks in *CYB18Q1*-sgRNA-L7 or an "A" insertion in *CYB18Q1*-sgRNA-L25. The black and red lines indicate the sgRNA and PAM sequence, respectively.

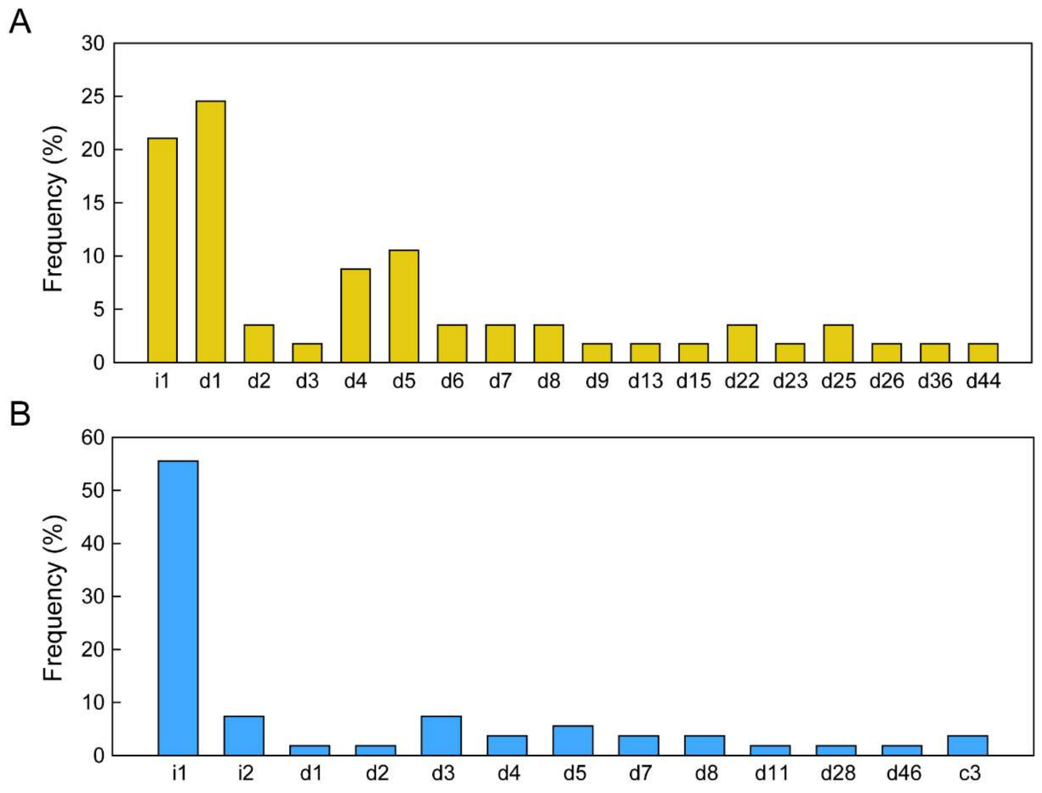

Figure S2. Summary of mutations in edited allele of two targets. A and B. Frequency of each mutation type for edited allele of (A) CYB18Q1 and (B) CYP92B14. i, insertion; d, deletion; c, combined mutation. d#, number of base pairs (bp) deleted from the target site; i#, number of bp inserted at target site; c#, number of bp combined mutations.

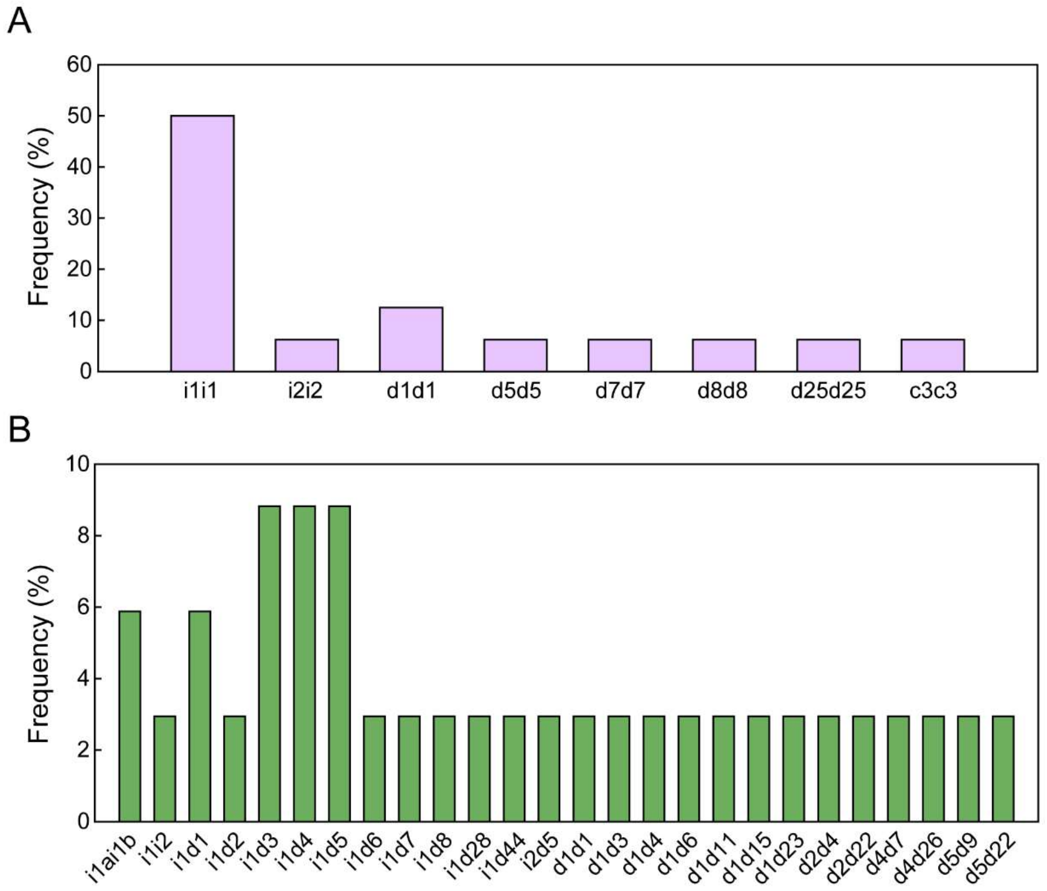

Figure S3. Frequency of each mutation type for homozygotes and bi-allelic mutations. A and B. Frequency of each mutation type for (A) homozygotes and (B) bi-allelic mutations. i, insertion; d, deletion; c, combined mutation. d#, number of base pairs (bp) deleted from the target site; i#, number of bp inserted at target site; i#a, same number of insertion at one site; i#b, same number of insertion of different nucleotide at the same site; c#, number of bp combined mutations.

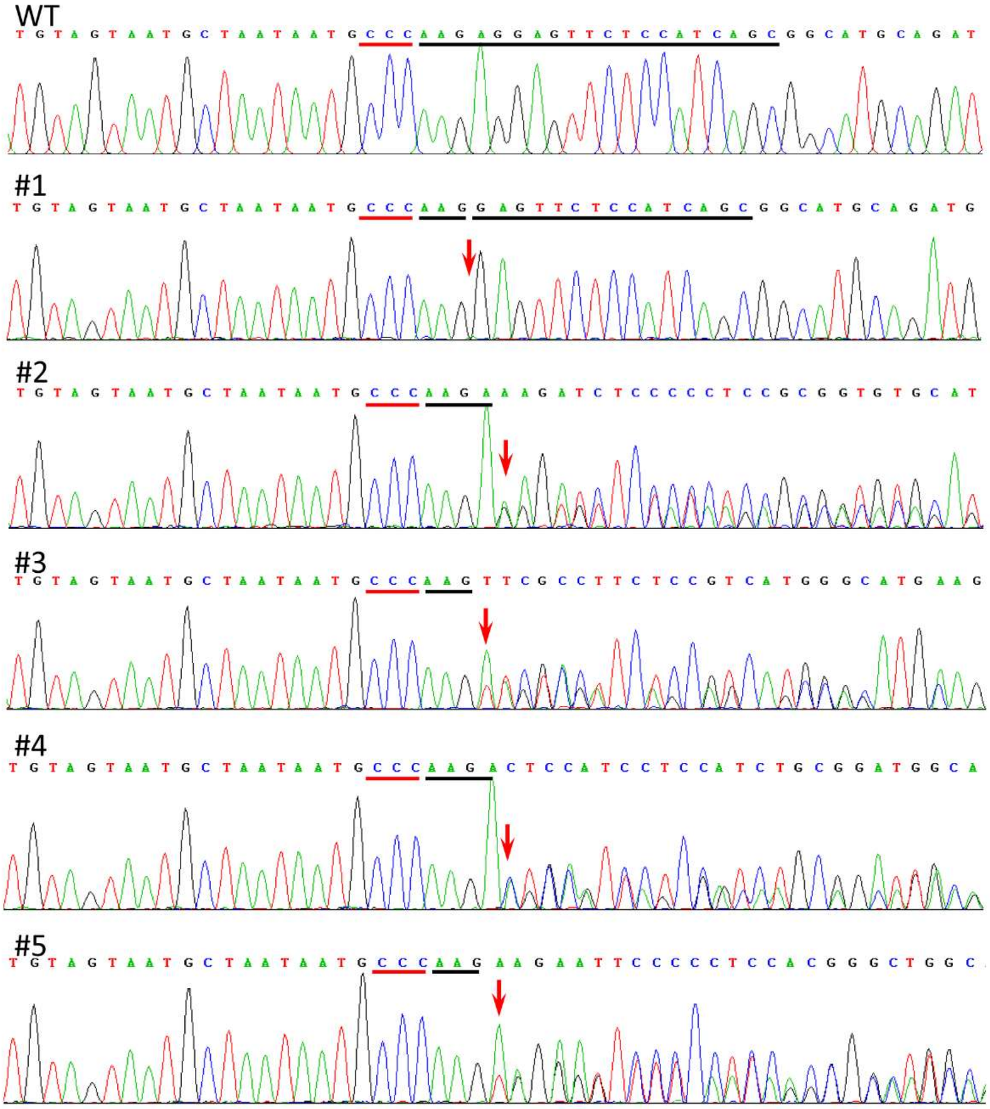

Figure S4. Off-target analysis in the putative off-target site 1 of *CYP92B14*-sgRNA. Sequencing chromatograms from WT and 5 transgenic hair root lines (#1-5) were shown. The red arrow indicates an "AG" deletion (in #1) or overlapping peaks (in #2, #3, #4 and #5). The black and red lines indicate the off-target sequences and PAM sequences in the off-target site 1, respectively. The sequencing chromatograms were the results sequenced from the reverse direction.

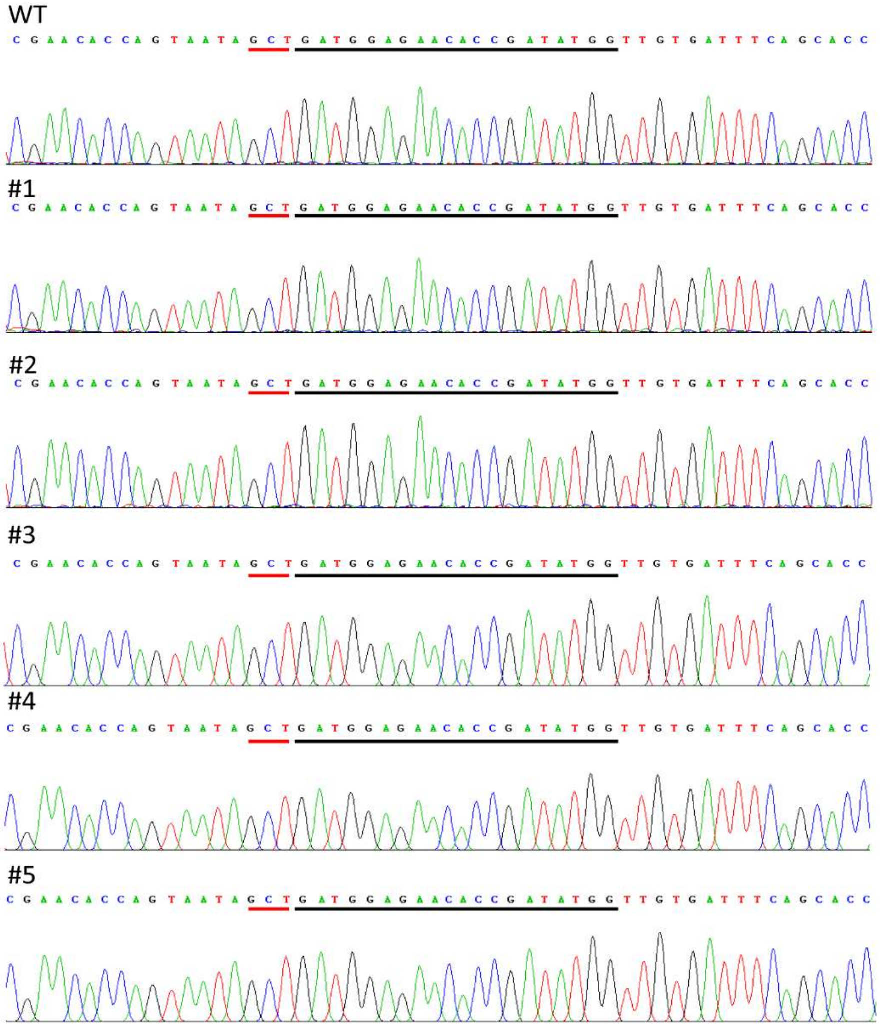

Figure S5. Off-target analysis in the putative off-target site 2 of *CYP92B14*-sgRNA. Sequencing chromatograms from WT and 5 transgenic hair root lines (#1-5) were shown. No mutation was detected in putative off-target site 2. The black and red lines indicate the off-target sequences and PAM sequences in the putative off-target site 2, respectively. The sequencing chromatograms were the results sequenced from the reverse direction.
