# Supplementary material for: CRISPR/Cas9-Mediated Efficient Targeted Mutagenesis in Sesame (Sesamum indicum L.)
Source: Front Plant Sci. 2022 Jul 11;13:935825. doi: 10.3389/fpls.2022.935825 (PMC9309882; doi:10.3389/fpls.2022.935825)
Supplement: Supplementary file 3 [file Data_Sheet_3.docx]

**Supplemental Data S1. Decoding results from *CYB18Q1*-sgRNA** **transgenic hairy roots.**

DNA was extracted from positive transgenic hairy roots. The DNA sequences containing the target sites were amplified by PCR and Sanger sequenced using specific internal primer. The sequencing results were decoded using the web-based tool DSDecode (<http://skl.scau.edu.cn/dsdecode/>). For the PCR amplicons contained complex mutations that could not be decoded in DSDecode, the PCR productions were subcloned into TA vector and then sequenced. Genotype of each allele was identified. The sgRNA targets are indicated with blue background and the PAM motif (NGG) is indicated with green background. The insertion and deletion of nucleotides are indicated with yellow background and red dashes.

***CYB18Q1*-sgRNA-L1**

**Allele1:****TGTTTCACCAAGTATGACATTGTCTTGGCAAATCGC****CCTCAGGCTTCTGTTGACCGGCGCTCACTTGG (WT)**

**Allele2:   TGTTTCACCAAGTATGAC------------------------------------CGGCGCTCACTTGG (deletion)**

**Reference: TGTTTCACCAAGTATGACATTGTCTTGGCAAATCGCCCTCAGGCTTCTGTTGACCGGCGCTCACTTGG**

**
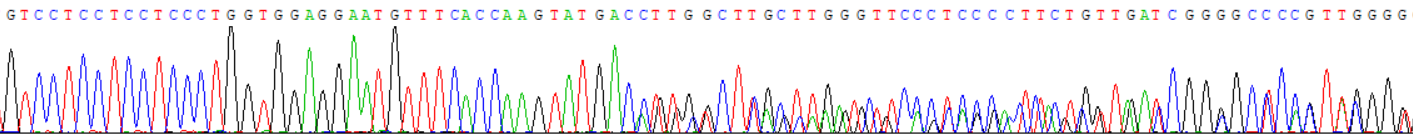
**

***CYB18Q1*-sgRNA-L2**

**Allele1:   GTCTTGGCAAATCGCCCTCA--GCTTCTGTTGACCGGCGCTCACTTGGGTTCAGCACCACCAGCGTA (deletion)**

**Allele2:   GTCTTGGCAAATCGCCCTCAAGGCTTCTGTTGACCGGCGCTCACTTGGGTTCAGCACCACCAGCGTA (insertion)**

**Reference: GTCTTGGCAAATCGCCCTCA-GGCTTCTGTTGACCGGCGCTCACTTGGGTTCAGCACCACCAGCGTAATCG**

**
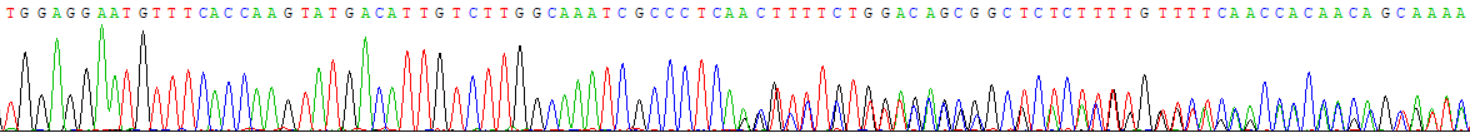
**

***CYB18Q1*-sgRNA-L3**

**Allele1:   GTCTTGGCAAATCGCCCTCAGGCTTCTGTTGACCGGCGCTCACTTGGGTTCAGCACCACCAGCGTAAT (WT)**

**Allele2:   GTCTTGGCAAATCGCCCTCAGGCTTCTGTTGACCGGCGCTCACTTGGGTTCAGCACCACCAGCGTAAT (WT)**

**Reference: GTCTTGGCAAATCGCCCTCAGGCTTCTGTTGACCGGCGCTCACTTGGGTTCAGCACCACCAGCGTAAT**

**
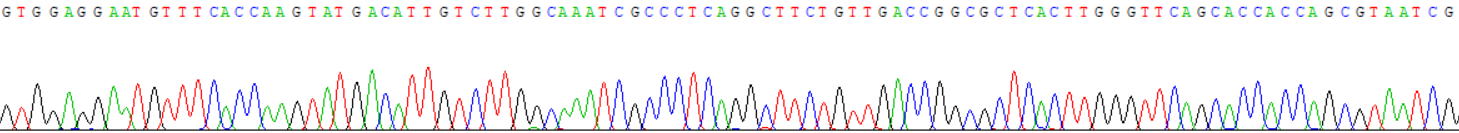
**

***CYB18Q1*-sgRNA-L4**

**Allele1:   GTCTTGGCAAATCGCCCTCA------CTGTTGACCGGCGCTCACTTGGGTTCAGCACCACCAGCGTA (deletion)**

**Allele2:   GTCTTGGCAAATCGCCCTCAGGGCTTCTGTTGACCGGCGCTCACTTGGGTTCAGCACCACCAGCGTA (insertion)**

**Reference: GTCTTGGCAAATCGCCCTCA-GGCTTCTGTTGACCGGCGCTCACTTGGGTTCAGCACCACCAGCGTA**

**
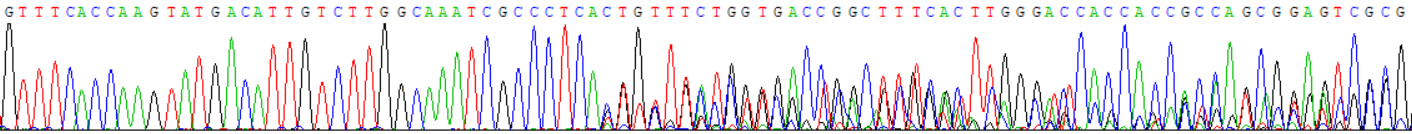
**

***CYB18Q1*-sgRNA-L5**

**Allele1:   AAGTATGACATTGTCTTGGC--------------------------GGCGCTCACTTGGGTTCAGC (deletion)**

**Allele2:   AAGTATGACATTGTCTTGGCAAATCGCCCTCA----TCTGTTGACCGGCGCTCACTTGGGTTCAGC (deletion)**

**Reference: AAGTATGACATTGTCTTGGCAAATCGCCCTCAGGCTTCTGTTGACCGGCGCTCACTTGGGTTCAGC**

**
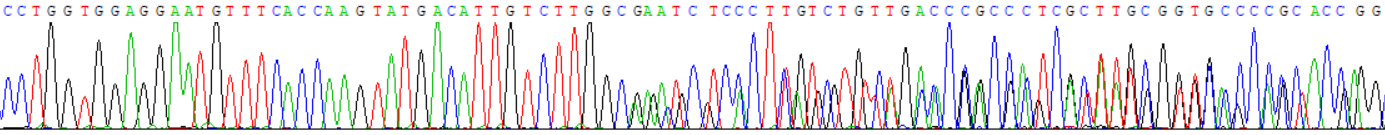
**

***CYB18Q1*-sgRNA-L6**

**Allele1:   AAATCGCCCTCA----TCTGTTGACCGGCGCTCACTTGGGTTCAGCACCACCAGCGTAATCG (deletion)**

**Allele2:   AAATCGCCCTCA-------GTTGACCGGCGCTCACTTGGGTTCAGCACCACCAGCGTAATCG (deletion)**

**Reference: AAATCGCCCTCAGGCTTCTGTTGACCGGCGCTCACTTGGGTTCAGCACCACCAGCGTAATCG**

**
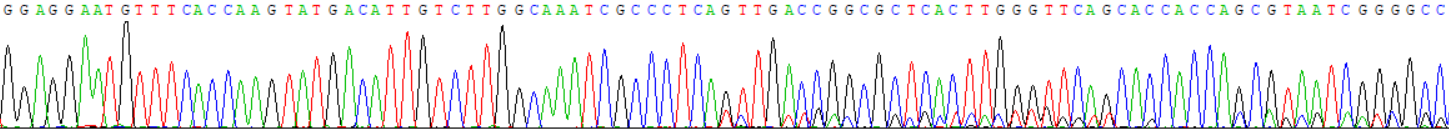
**

***CYB18Q1*-sgRNA-L7**

**Allele1:   GTCTTGGCAAATCGCCCTCA-GCTTCTGTTGACCGGCGCTCACTTGGGTTCAGCACCACCAGC (deletion)**

**Allele2:   GTCTTGGCAAATCGCCCTCA------TGTTGACCGGCGCTCACTTGGGTTCAGCACCACCAGC (deletion)**

**Reference: GTCTTGGCAAATCGCCCTCAGGCTTCTGTTGACCGGCGCTCACTTGGGTTCAGCACCACCAGC 

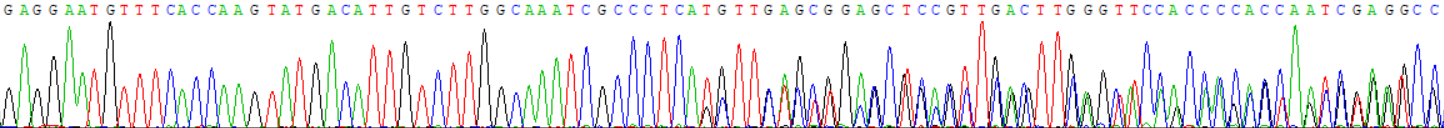
**

***CYB18Q1*-sgRNA-L8**

**Allele1:   TGACATTGTCTTGGCAAATCGCC--------TTCTGTTGACCGGCGCTCACTTGGGTTCAGCAC (deletion)**

**Allele2:   TGACATTGTCTTGGCAAATCGCCCTCATGGCTTCTGTTGACCGGCGCTCACTTGGGTTCAGCAC (insertion)**

**Reference: TGACATTGTCTTGGCAAATCGCCCTCA-GGCTTCTGTTGACCGGCGCTCACTTGGGTTCAGCAC**

**
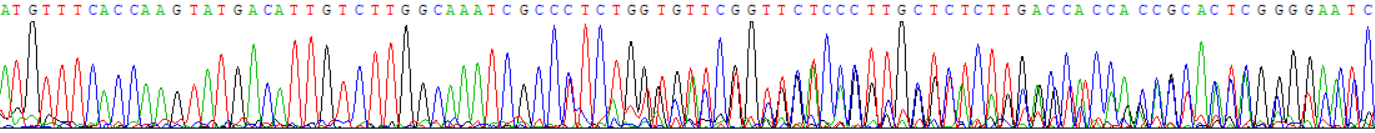
**

***CYB18Q1*-sgRNA-L9**

**Allele1:   TTGTCTTGGCAAATCGCC-(44-bp deletion)AGCGTAATCGGGG (deletion)**

**Allele2:   TTGTCTTGGCAAATCGCCCTCATGGCTTCTGTTGACCGGCGCTCACTTGGGTTCAGCACCACCAGC (insertion)**

**Reference: TTGTCTTGGCAAATCGCCCTCA-GGCTTCTGTTGACCGGCGCTCACTTGGGTTCAGCACCACCAGC**

**
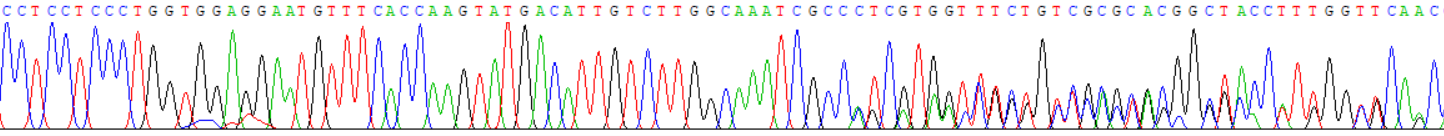
**

***CYB18Q1*-sgRNA-L10**

**Allele1:   GTCTTGGCAAATCGCCCTCA-GCTTCTGTTGACCGGCGCTCACTTGGGTTCAGCACCACCAGCGTAA (deletion)**

**Allele2:   GTCTTGGCAAATCGCCCTCA---TTCTGTTGACCGGCGCTCACTTGGGTTCAGCACCACCAGCGTAA (deletion)**

**Reference: GTCTTGGCAAATCGCCCTCAGGCTTCTGTTGACCGGCGCTCACTTGGGTTCAGCACCACCAGCGTAA**

**
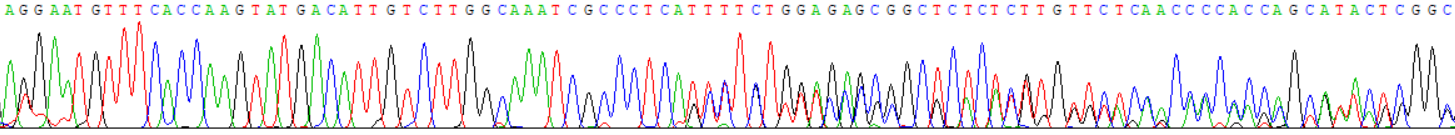
**

***CYB18Q1*-sgRNA-L11**

**Allele1:   GTCTTGGCAAATCGCCCTCAGGCTTCTGTTGACCGGCGCTCACTTGGGTTCAGCACCACCAGCGTAAT (WT)**

**Allele2:   GTCTTGGCAAATCGCCCTCAGGCTTCTGTTGACCGGCGCTCACTTGGGTTCAGCACCACCAGCGTAAT (WT)**

**Reference: GTCTTGGCAAATCGCCCTCAGGCTTCTGTTGACCGGCGCTCACTTGGGTTCAGCACCACCAGCGTAAT**

**
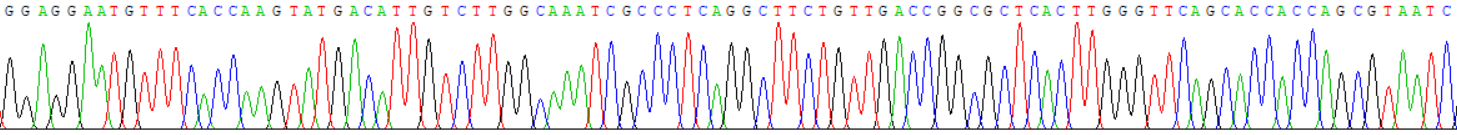
**

***CYB18Q1*-sgRNA-L12**

**Allele1:   ACATTGTCTTGGCAAATCGC--------TTCTGTTGACCGGCGCTCACTTGGGTTCAG (deletion)**

**Allele2:   ACATTGTCTTGGCAAATCGC--------TTCTGTTGACCGGCGCTCACTTGGGTTCAG (deletion)**

**Reference: ACATTGTCTTGGCAAATCGCCCTCAGGCTTCTGTTGACCGGCGCTCACTTGGGTTCAG**

**
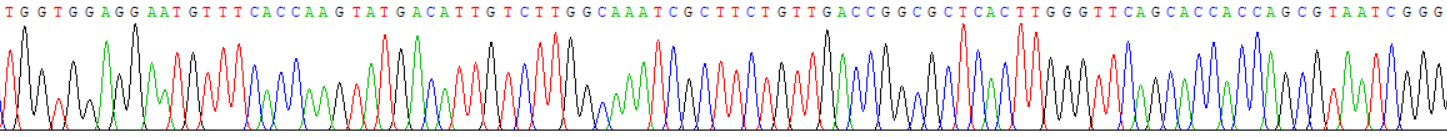
**

***CYB18Q1*-sgRNA-L13**

**Allele1:   TCTTGGCAAATCG****CCCTCA-GCTTCTGTTGACCGGCGCTCACTTGGGTTCAG (deletion)**

**Allele2:   TCTTGGCAAATCGCCCTCA-GCTTCTGTTGACCGGCGCTCACTTGGGTTCAG (deletion)**

**Reference: TCTTGGCAAATCGCCCTCAGGCTTCTGTTGACCGGCGCTCACTTGGGTTCAG**

**
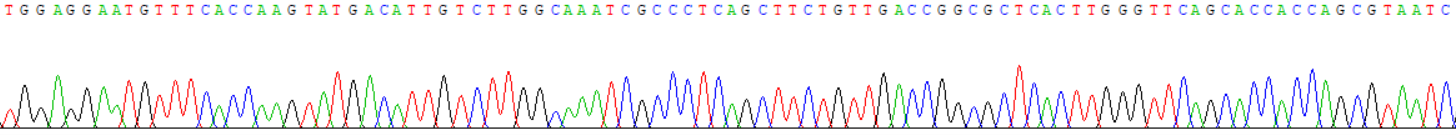
**

***CYB18Q1*-sgRNA-L14**

**Allele1:   GTCTTGGCAAATCGCCCTCA-------TGTTGACCGGCGCTCACTTGGGTTCAGCACCACCAG (deletion)**

**Allele2:   GTCTTGGCAAATCGCCCTCAAGGCTTCTGTTGACCGGCGCTCACTTGGGTTCAGCACCACCAG (insertion)**

**Reference: GTCTTGGCAAATCGCCCTCA-GGCTTCTGTTGACCGGCGCTCACTTGGGTTCAGCACCACCAG**

**
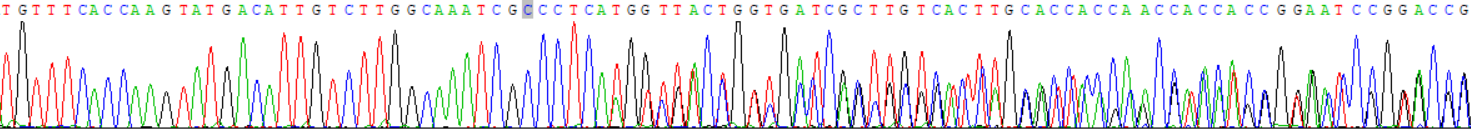
**

***CYB18Q1*-sgRNA-L15**

**Allele1:   CTTGGCAAATCGCCCTCA----------------------CTTGGGTTCAGCACCACCAGCGT (deletion)**

**Allele2:   CTTGGCAAATCGCCCTCA--CTTCTGTTGACCGGCGCTCACTTGGGTTCAGCACCAC****CAGCGT (deletion)**

**Reference: CTTGGCAAATCGCCCTCAGGCTTCTGTTGACCGGCGCTCACTTGGGTTCAGCACCACCAGCGT**

**
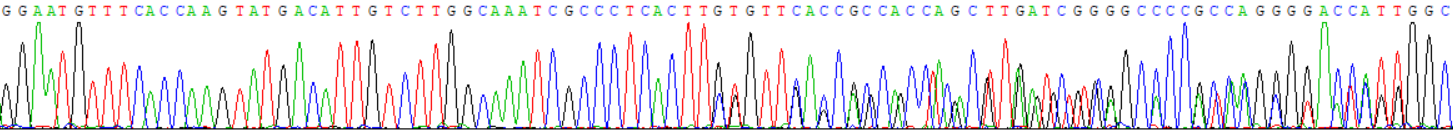
**

***CYB18Q1*-sgRNA-L16**

**Allele1:   TGTCTTGGCAAATCGCCCTCA-GCTTCTGTTGACCGGCGCTCACTTGGGTTCAGCACCACCAG (deletion)**

**Allele2:   TGTCTTGGCAAATCGCCCTC-GGCTTCTGTTGACCGGCGCTCACTTGGGTTCAGCACCACCAG (deletion)**

**Reference: TGTCTTGGCAAATCGCCCTCAGGCTTCTGTTGACCGGCGCTCACTTGGGTTCAGCACCACCAG**

**
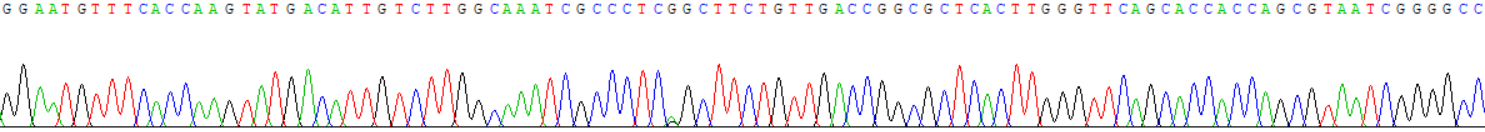
**

***CYB18Q1*-sgRNA-L17**

**Allele1:   CCAAGTATGACATTGTCTTG-------------------------ACCGGCGCTCACTTG (deletion)**

**Allele2:   CCAAGTATGACATTGTCTTG-------------------------ACCGGCGCTCACTTG (deletion)**

**Reference: CCAAGTATGACATTGTCTTGGCAAATCGCCCTCAGGCTTCTGTTGACCGGCGCTCACTTG**

**
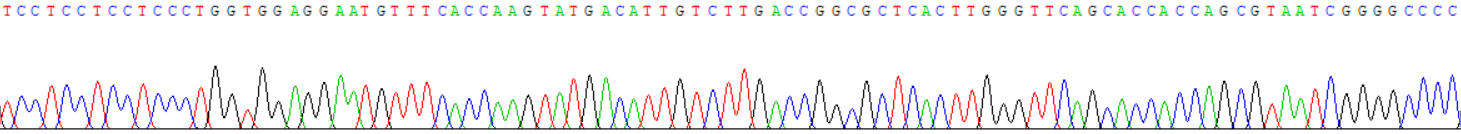
**

***CYB18Q1*-sgRNA-L18**

**Allele1:   GTCTTGGCAAATCGCCCTCA-----CTGTTGACCGGCGCTCACTTGGGTTCAGCACCACCAGC (deletion)**

**Allele2:   GTCTTGGCAAATCGCCCTCA-----CTGTTGACCGGCGCTCACTTGGGTTCAGCACCACCAGC (deletion)**

**Reference: GTCTTGGCAAATCGCCCTCAGGCTTCTGTTGACCGGCGCTCACTTGGGTTCAGCACCACCAGC**

**
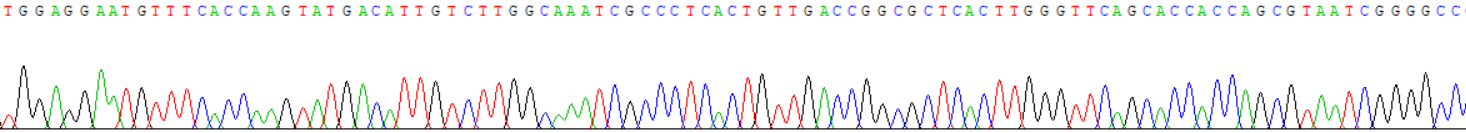
**

***CYB18Q1*-sgRNA-L19**

**Allele1:   AAGTATGACATTGTCTTGGC---------------TTCTGTTGACCGGCGCTCACTTGGGTTCA (deletion)**

**Allele2:   AAGTATGACATTGTCTTGGCAAATCGCCCTCA-GCTTCTGTTGACCGGCGCTCACTTGGGTTCA (deletion)**

**Reference: AAGTATGACATTGTCTTGGCAAATCGCCCTCAGGCTTCTGTTGACCGGCGCTCACTTGGGTTCA**

**
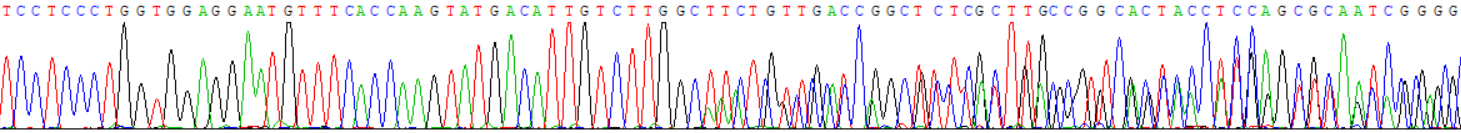
**

***CYB18Q1*-sgRNA-L20**

**Allele1:   TCTTGGCAAATCGCCCTCA--GCTTCTGTTGACCGGCGCTCACTTGGGTTCAGCACCACCAG (deletion)**

**Allele2:   TCTTGGCAAATCGCCCTCAGGGCTTCTGTTGACCGGCGCTCACTTGGGTTCAGCACCACCAG (insertion)**

**Reference: TCTTGGCAAATCGCCCTCA-GGCTTCTGTTGACCGGCGCTCACTTGGGTTCAGCACCACCAG**

**
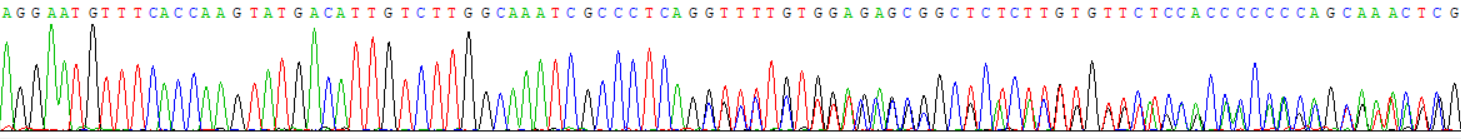
**

***CYB18Q1*-sgRNA-L21**

**Allele1:   GTCTTGGCAAATCGCCCTCAGGGCTTCTGTTGACCGGCGCTCACTTGGGTTCAGCACCACCAGCGT (WT)**

**Allele2:   GTCTTGGCAAATCGCCCTCAGGCTTCTGTTGACCGGCGCTCACTTGGGTTCAGCACCACCAGCGTA (WT)**

**Reference: GTCTTGGCAAATCGCCCTCAGGCTTCTGTTGACCGGCGCTCACTTGGGTTCAGCACCACCAGCGTA**

**
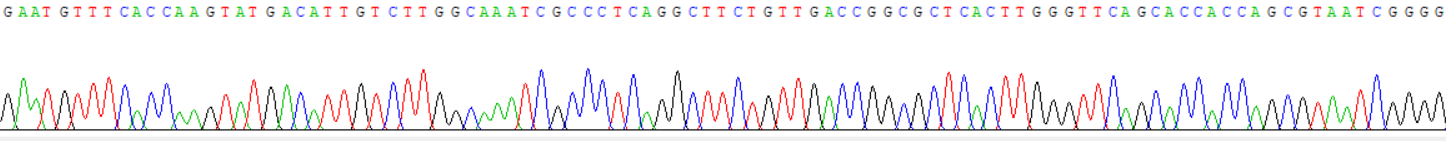
**

***CYB18Q1*-sgRNA-L22**

**Allele1:   TCTTGGCAAATCGCCCTCA-GCTTCTGTTGACCGGCGCTCACTTGGGTTCAGCACCACCAGCGT (deletion)**

**Allele2:   TCTTGGCAAATCGCCCTCA-GCTTCTGTTGACCGGCGCTCACTTGGGTTCAGCACCACCAGCGT (deletion)**

**Reference: TCTTGGCAAATCGCCCTCAGGCTTCTGTTGACCGGCGCTCACTTGGGTTCAGCACCACCAGCGT**

**
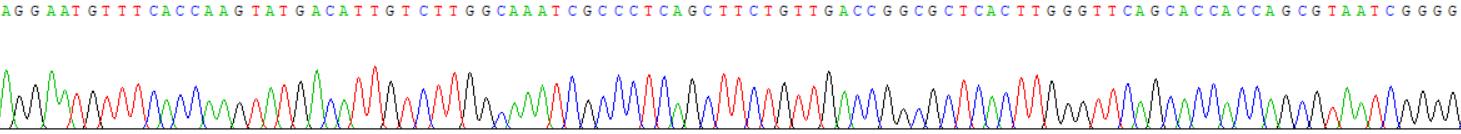
**

***CYB18Q1*-sgRNA-L23**

**Allele1:   ATGTTTCACCAAGTATGACA-----------------------GCTTCTGTTGACCGGCGCTCA (deletion)**

**Allele2:   ATGTTTCACCAAGTATGACATTGTCTTGGCAAATCGCCCTCA-GCTTCTGTTGACCGGCGCTCA (deletion)**

**Reference: ATGTTTCACCAAGTATGACATTGTCTTGGCAAATCGCCCTCAGGCTTCTGTTGACCGGCGCTCA**

**
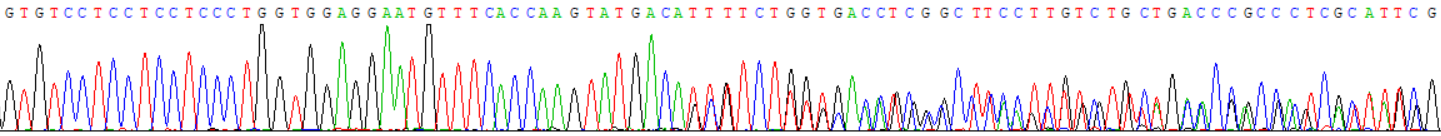
**

***CYB18Q1*-sgRNA-L24**

**Allele1:   TTGTCTTGGCAAATCGCCCT---------GTTGACCGGCGCTCACTTGGGTTCAGCACCACCAG (deletion)**

**Allele2:   TTGTCTTGGCAAATCGCCCTCA-----CTGTTGACCGGCGCTCACTTGGGTTCAGCACCACCAG (deletion)**

**Reference: TTGTCTTGGCAAATCGCCCTCAGGCTTCTGTTGACCGGCGCTCACTTGGGTTCAGCACCACCAG**

**
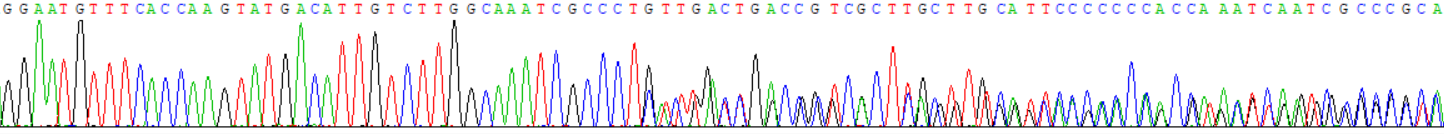
**

***CYB18Q1*-sgRNA-L25**

**Allele1:   GTCTTGGCAAATCGCCCTCAAGGCTTCTGTTGACCGGCGCTCACTTGGGTTCAGCACCACCAG (insertion)**

**Allele2:   GTCTTGGCAAATCGCCCTCAAGGCTTCTGTTGACCGGCGCTCACTTGGGTTCAGCACCACCAG (insertion)**

**Reference: GTCTTGGCAAATCGCCCTCA-GGCTTCTGTTGACCGGCGCTCACTTGGGTTCAGCACCACCAG**

**
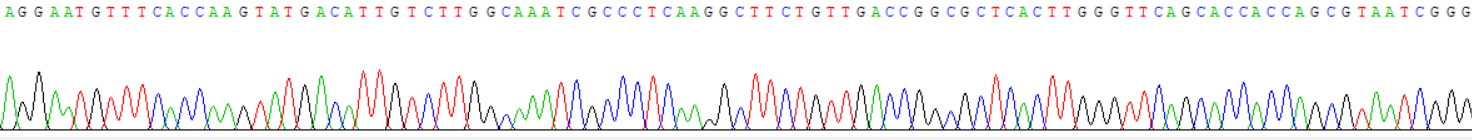
**

***CYB18Q1*-sgRNA-L26**

**Allele1:   GTCTTGGCAAATCGCCCTCA--GCTTCTGTTGACCGGCGCTCACTTGGGTTCAGCACCAC (deletion)**

**Allele2:   GTCTTGGCAAATCGCCCTCATGGCTTCTGTTGACCGGCGCTCACTTGGGTTCAGCACCAC (insertion)**

**Reference: GTCTTGGCAAATCGCCCTCA-GGCTTCTGTTGACCGGCGCTCACTTGGGTTCAGCACCAC**

**
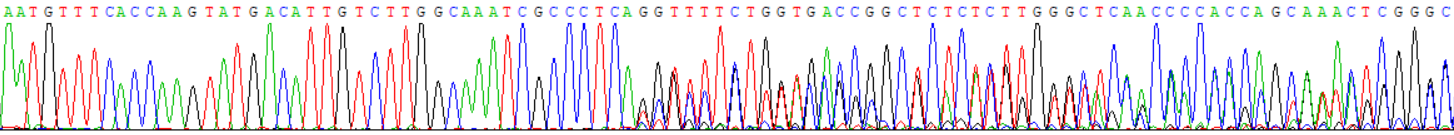
**

***CYB18Q1*-sgRNA-L27**

**Allele1:   TGTCTTGGCAAATCGCCCTCAAGGCTTCTGTTGACCGGCGCTCACTTGGGTTCAGCACCAC (insertion)**

**Allele2:   TGTCTTGGCAAATCGCCCTCA--GCTTCTGTTGACCGGCGCTCACTTGGGTTCAGCACCAC (deletion)**

**Allele3:   TGTCTTGGCAAATCGCCCTCA------CTGTTGACCGGCGCTCACTTGGGTTCAGCACCAC (deletion)**

**Reference: TGTCTTGGCAAATCGCCCTCA-GGCTTCTGTTGACCGGCGCTCACTTGGGTTCAGCACCAC**

**
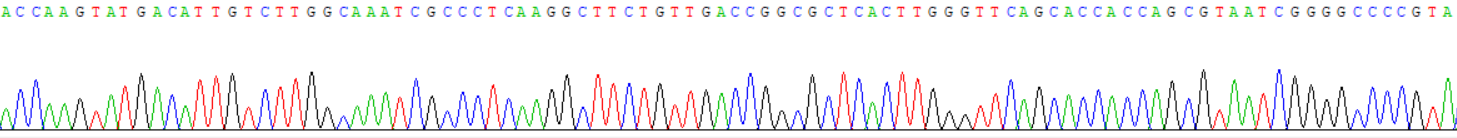
**

**
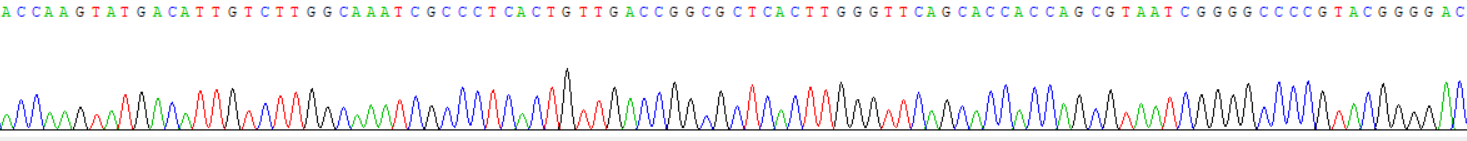

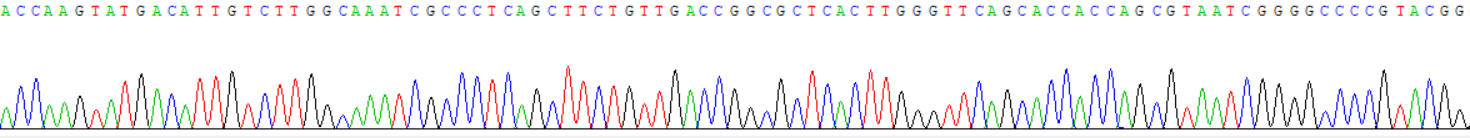
**

***CYB18Q1*-sgRNA-L28**

**Allele1:   TGTCTTGGCAAATCGCCCTC-----TTCTGTTGACCGGCGCTCACTTGGGTTCAGCACCAC (deletion)**

**Allele2:   TGTCTTGGCAAATCGCCCTCAAGGCTTCTGTTGACCGGCGCTCACTTGGGTTCAGCACCAC (insertion)**

**Reference: TGTCTTGGCAAATCGCCCTCA-GGCTTCTGTTGACCGGCGCTCACTTGGGTTCAGCACCAC**

**
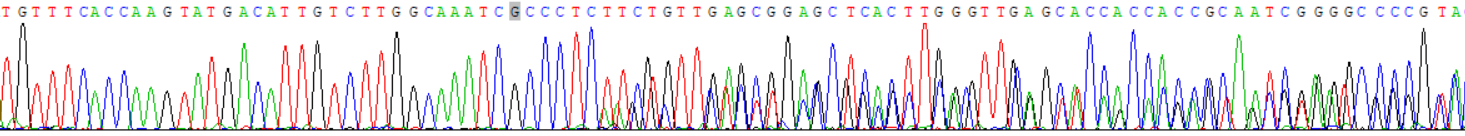
**

***CYB18Q1*-sgRNA-L29**

**Allele1:   TGTCTTGGCAAATCGCCCTCATGGCTTCTGTTGACCGGCGCTCACTTGGGTTCAGCACCAC (insertion)**

**Allele2:   TGTCTTGGCAAATCGCCCTCA-----TCTGTTGACCGGCGCTCACTTGGGTTCAGCACCAC (deletion)**

**Reference: TGTCTTGGCAAATCGCCCTCA-GGCTTCTGTTGACCGGCGCTCACTTGGGTTCAGCACCAC**

**
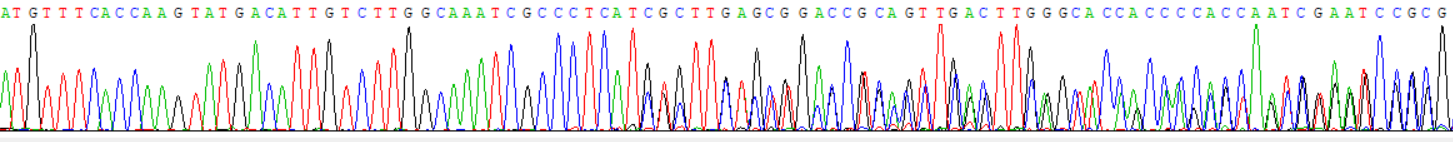
**

***CYB18Q1*-sgRNA-L30**

**Allele1:   TGTCTTGGCAAATCGCCCTCA-GCTTCTGTTGACCGGCGCTCACTTGGGTTCAGCACCAC (deletion)**

**Allele2:   TGTCTTGGCAAATCGCCCTCA----TCTGTTGACCGGCGCTCACTTGGGTTCAGCACCAC (deletion)**

**Reference: TGTCTTGGCAAATCGCCCTCAGGCTTCTGTTGACCGGCGCTCACTTGGGTTCAGCACCAC**

**
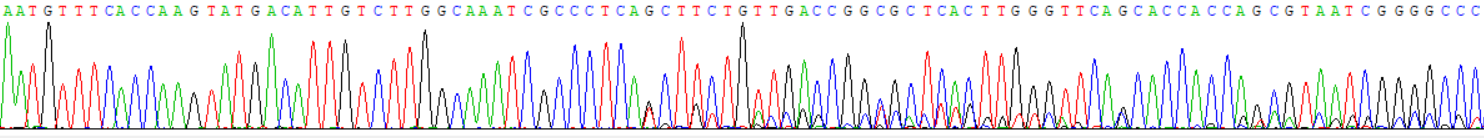
**

***CYB18Q1*-sgRNA-L31**

**Allele1:   TTGTCTTGGCAAATCGCCCTCAGGCTTCTGTTGACCGGCGCTCACTTGGGTTCAGCACCA (WT)**

**Allele2:   TTGTCTTGGCAAATCGCCCT-------------ACCGGCGCTCACTTGGGTTCAGCACCA (deletion)**

**Reference: TTGTCTTGGCAAATCGCCCTCAGGCTTCTGTTGACCGGCGCTCACTTGGGTTCAGCACCA**

**
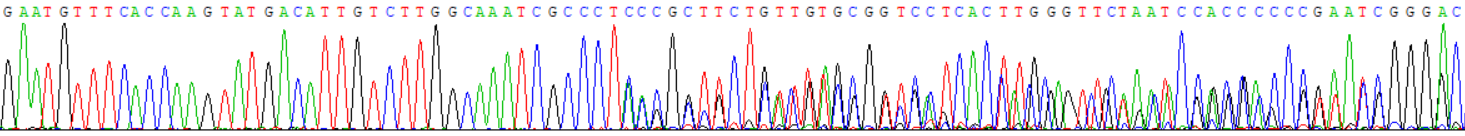
**

***CYB18Q1*-sgRNA-L32**

**Allele1:   GTCTTGGCAAATCGCCCTCA-----CTGTTGACCGGCGCTCACTTGGGTTCAGCACCACCA (deletion)**

**Allele2:   GTCTTGGCAAATCGCCCTCA----------------------CTTGGGTTCAGCACCACCA (deletion)**

**Reference: GTCTTGGCAAATCGCCCTCAGGCTTCTGTTGACCGGCGCTCACTTGGGTTCAGCACCACCA**

**
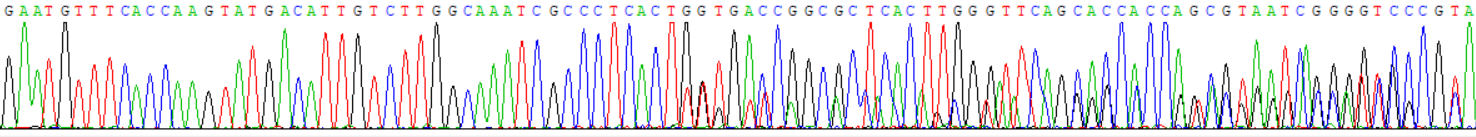
**
