# Supplementary material for: CRISPR/Cas9-Mediated Efficient Targeted Mutagenesis in Sesame (Sesamum indicum L.)
Source: Front Plant Sci. 2022 Jul 11;13:935825. doi: 10.3389/fpls.2022.935825 (PMC9309882; doi:10.3389/fpls.2022.935825)

**Supplemental Data S2. Decoding results from *CYP92B14*-sgRNA** **transgenic hairy roots.**

DNA was extracted from positive transgenic hairy roots. The DNA sequences containing the target sites were amplified by PCR and Sanger sequenced using specific internal primer. The sequencing results were decoded using the web-based tool DSDecode (<http://skl.scau.edu.cn/dsdecode/>). For the PCR amplicons contained complex mutations that could not be decoded in DSDecode, the PCR productions were subcloned into TA vector and then sequenced. Genotype of each allele was identified. The sgRNA targets are indicated with blue background and the PAM motif (NGG) is indicated with green background. The insertion and deletion of nucleotides are indicated with yellow background and red dashes. The sequencing chromatograms at the *CYP92B14*-sgRNA region were the results sequenced from the reverse direction.

***CYP92B14*-sgRNA-L1**

**Allele1:   AATGCTAATAATGCCCAAGC-------TCCATCAGCGGCATCCAGAGGTTGTGGCAGAG (deletion)**

**Allele2:   AATGCTAATAATGCCCAAGC-------TCCATCAGCGGCATCCAGAGGTTGTGGCAGAG (deletion)**

**Reference: AATGCTAATAATGCCCAAGCGGAGTTCTCCATCAGCGGCATCCAGAGGTTGTGGCAGAG**

**
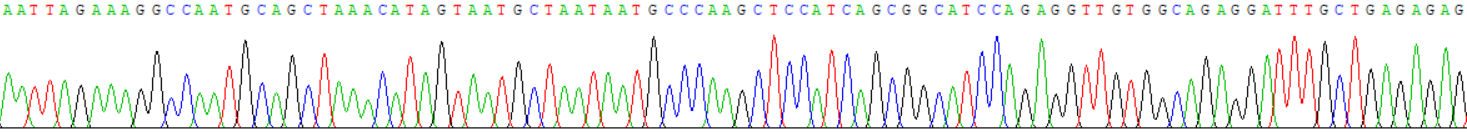
**

***CYP92B14*-sgRNA-L2**

**Allele1:   TAATGCTAATAATGCCCAAG----AGTTCTCCATCAGCGGCATCCAGAGGTTGTGGCAGAG (deletion)**

**Allele2:   TAATGCTAATAATGCCCAAGTCGGAGTTCTCCATCAGCGGCATCCAGAGGTTGTGGCAGAG (insertion)**

**Reference: TAATGCTAATAATGCCCAAG-CGGAGTTCTCCATCAGCGGCATCCAGAGGTTGTGGCAGAG**

**
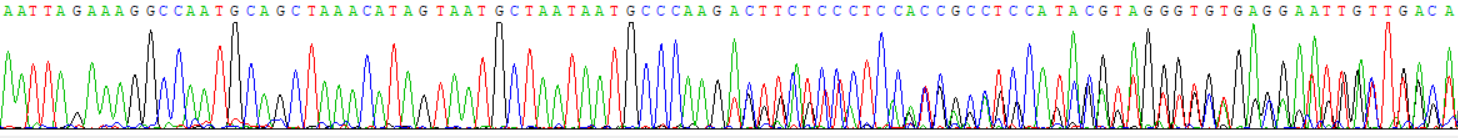
**

***CYP92B14*-sgRNA-L3**

**Allele1:   TAATGCTAATAATGCCCAAG------TTCTCCATCAGCGGCATCCAGAGGTTGTGGCAGA (deletion)**

**Allele2:   TAATGCTAATAATGCCCAAGTCGGAGTTCTCCATCAGCGGCATCCAGAGGTTGTGGCAGA (insertion)**

**Reference: TAATGCTAATAATGCCCAAG-CGGAGTTCTCCATCAGCGGCATCCAGAGGTTGTGGCAGA**

**
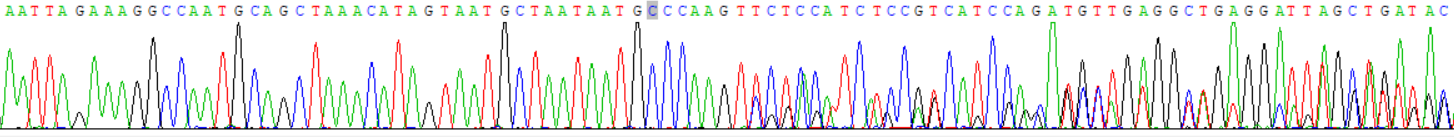
**

***CYP92B14*-sgRNA-L4**

**Allele1:   TAATGCTAATAATGCCCAAGACGGAGTTCTCCATCAGCGGCATCCAGAGGTTGTGGCAGA (insertion)**

**Allele2:   TAATGCTAATAATGCCCAAGTCGGAGTTCTCCATCAGCGGCATCCAGAGGTTGTGGCAGA (insertion)**

**Reference: TAATGCTAATAATGCCCAAG-CGGAGTTCTCCATCAGCGGCATCCAGAGGTTGTGGCAGA**

**
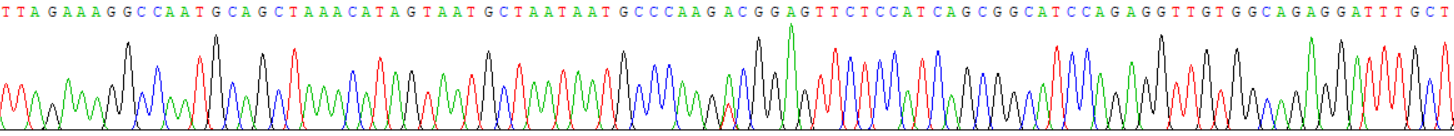
**

***CYP92B14*-sgRNA-L5**

**Allele1:   TAATGCTAATAATG****CCCAAGTCGGAGTTCTCCATCAGCGGCATCCAGAGGTTGTGGCAGA (insertion)**

**Allele2:   TAATGCTAATAATGCCCAAGTCGGAGTTCTCCATCAGCGGCATCCAGAGGTTGTGGCAGA (insertion)**

**Reference: TAATGCTAATAATGCCCAAG-CGGAGTTCTCCATCAGCGGCATCCAGAGGTTGTGGCAGA**

**
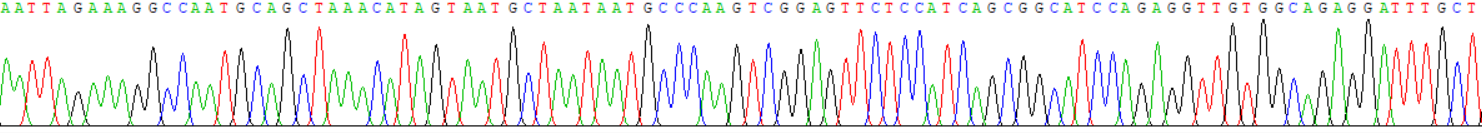
**

***CYP92B14*-sgRNA-L6**

**Allele1:   TAATGCTAATAATGCCCAAG------TTCTCCATCAGCGGCATCCAGAGGTTGTGGCAGA (deletion)**

**Allele2:   TAATGCTAATAATGCCCAAGTCGTACTTCTCCGTCAGCGGCATCCAGAGGTTGTGGCAGA (insertion)**

**Reference: TAATGCTAATAATGCCCAAG-CGGAGTTCTCCATCAGCGGCATCCAGAGGTTGTGGCAGA**


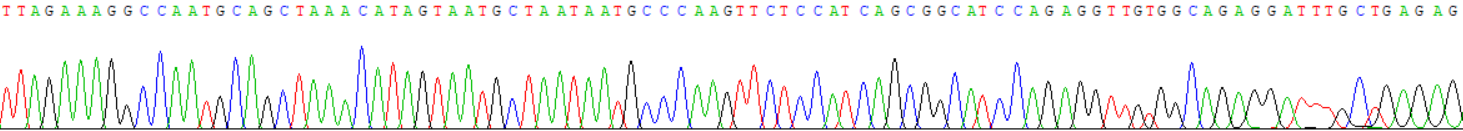


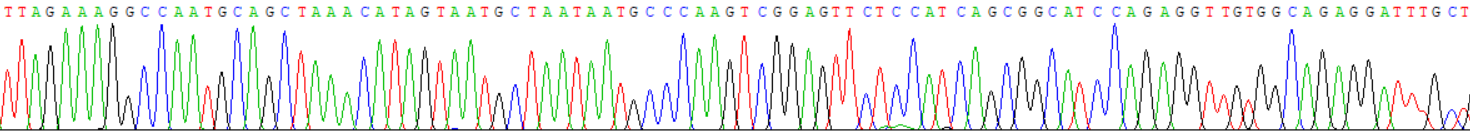


***CYP92B14*-sgRNA-L7**

**Allele1:   TAATGCTAATAATG****CCCAAG----AGTTCTCCATCAGCGGCATCCAGAGGTTGTGGCAGAGG (deletion)**

**Allele2:   TAATGCTAATAATGCCCAAGTCGGAGTTCTCCATCAGCGGCATCCAGAGGTTGTGGCAGAGG (insertion)**

**Reference: TAATGCTAATAATGCCCAAG-CGGAGTTCTCCATCAGCGGCATCCAGAGGTTGTGGCAGAGG**

**
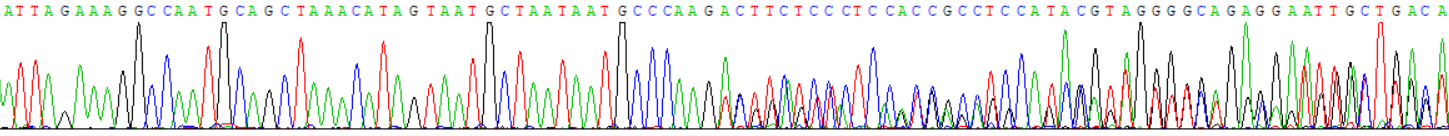
**

***CYP92B14*-sgRNA-L8**

**Allele1:   GCCAATGCAGCTAAACATAGTAATGCTAATAATGCCCAAGCGGAGTTCTCCATCAGCGGCATC (WT)**

**Allele2:   GCCAATGCAGCTAAACATAG(46-bp deletion)AGGTTGTGGCAGAGG (deletion)**

**Reference: GCCAATGCAGCTAAACATAGTAATGCTAATAATGCCCAAGCGGAGTTCTCCATCAGCGGCATC**

**
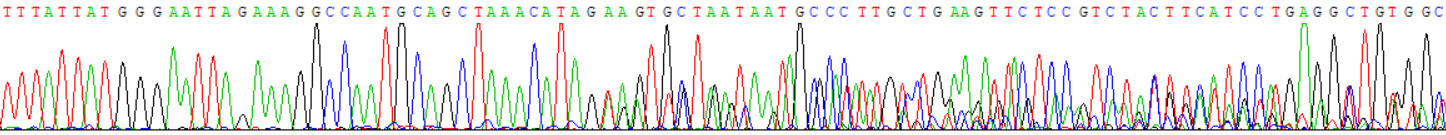
**

***CYP92B14*-sgRNA-L9**

**Allele1:   TAATGCTAATAATGCCC-----GGAGTTCTCCATCAGCGGCATCCAGAGGTTGTGGCAGA (deletion)**

**Allele2:   TAATGCTAATAATGCCCAAGTCGGAGTTCTCCATCAGCGGCATCCAGAGGTTGTGGCAGA (insertion)**

**Reference: TAATGCTAATAATGCCCAAG-CGGAGTTCTCCATCAGCGGCATCCAGAGGTTGTGGCAGA**

**
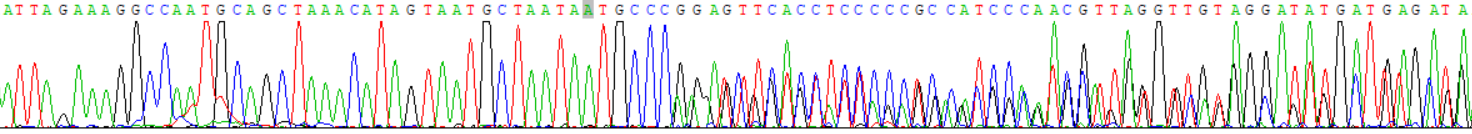
**

***CYP92B14*-sgRNA-L10**

**Allele1:   TAATGCTAATAATGCCCAAGTCGGAGTTCTCCATCAGCGGCATCCAGAGGTTGTGGCA (insertion)**

**Allele2:   TAATGCTAATAATGCCCAAGTCGGAGTTCTCCATCAGCGGCATCCAGAGGTTGTGGCA (insertion)**

**Reference: TAATGCTAATAATGCCCAAG-CGGAGTTCTCCATCAGCGGCATCCAGAGGTTGTGGCA**

**
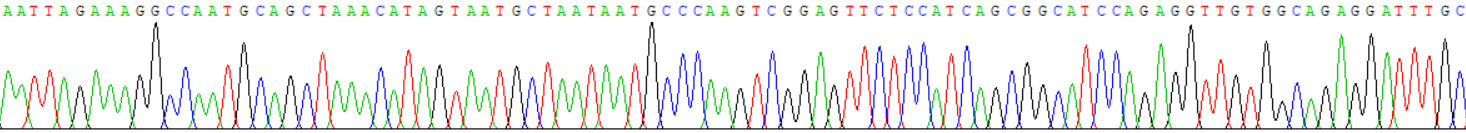
**

***CYP92B14*-sgRNA-L11**

**Allele1:   TGCTAATAATGCCC-----------TCCATCAGCGGCATCCAGAGGTTGTGGCAGAG (deletion)**

**Allele2:   TGCTAATAATGCCCAAG-GGAGTTCTCCATCAGCGGCATCCAGAGGTTGTGGCAGAG (deletion)**

**Reference: TGCTAATAATGCCCAAGCGGAGTTCTCCATCAGCGGCATCCAGAGGTTGTGGCAGAG**

**
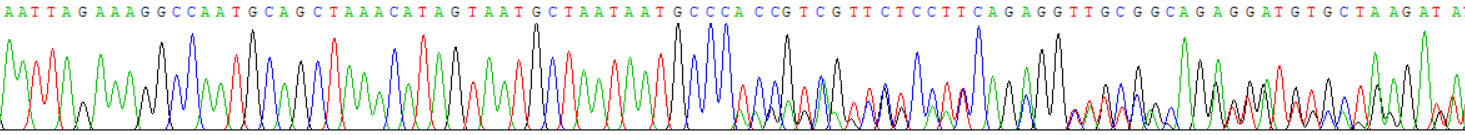
**

***CYP92B14*-sgRNA-L12**

**Allele1:   CTAATAATGCCCAAG-GCGGAGTTCTCCATCAGCGGCATCCAGAGGTTGTGGCAGAG (insertion)**

**Allele2:   CTAATAATGCCCAAGTCCGGAGTTCTCCATCAGCGGCATCCAGAGGTTGTGGCAGAG (insertion)**

**Reference: CTAATAATGCCCAAG--CGGAGTTCTCCATCAGCGGCATCCAGAGGTTGTGGCAGAG**

**
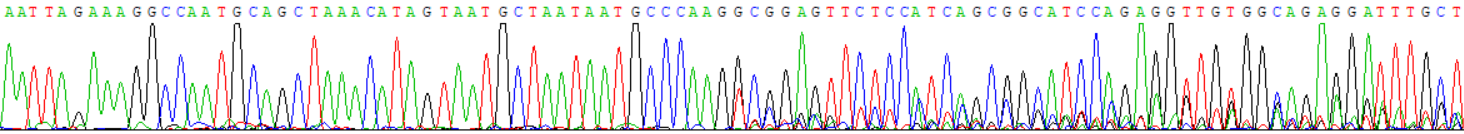
**

***CYP92B14*-sgRNA-L13**

**Allele1:   TGCTAATAATGCCCAAGCGGAGTTCTCCATCAGCGGCATCCAGAGGTTGTGGCAGAG (WT)**

**Allele2:   TGCTAATAATGCCCAAGCGGAGTTCTCCATCAGCGGCATCCAGAGGTTGTGGCAGAG (WT)**

**Reference: TGCTAATAATGCCCAAGCGGAGTTCTCCATCAGCGGCATCCAGAGGTTGTGGCAGAG**

**
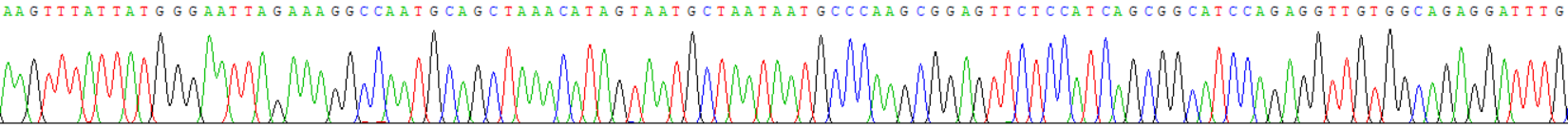
**

***CYP92B14*-sgRNA-L14**

**Allele1:   ATAATGCCCAAG-------TTCTCCATCAGCGGCATCCAGAGGTTGTGGCAGAGGAT (deletion)**

**Allele2:   ATAATGCCCAAGGTCGGAGTTCTCCATCAGCGGCATCCAGAGGTTGTGGCAGAGGAT (insertion)**

**Reference: ATAATGCCCAAG--CGGAGTTCTCCATCAGCGGCATCCAGAGGTTGTGGCAGAGGAT**

**
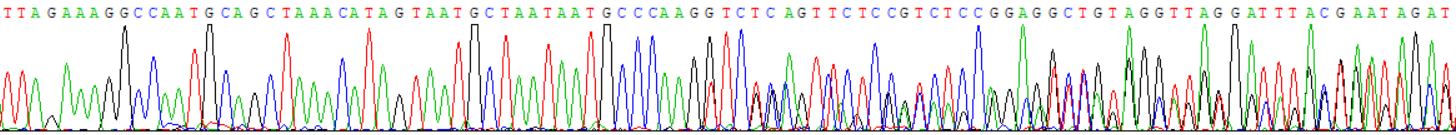
**

***CYP92B14*-sgRNA-L15**

**Allele1:   CTAATAATGCCC----GGAGTTCTCCATCAGCGGCATCCAGAGGTTGTGGCAGAG (deletion)**

**Allele2:   CTAATAATGCCCAAG--GAGTTCTCCATCAGCGGCATCCAGAGGTTGTGGCAGAG (deletion)**

**Reference: CTAATAATGCCCAAGCGGAGTTCTCCATCAGCGGCATCCAGAGGTTGTGGCAGAG**

**
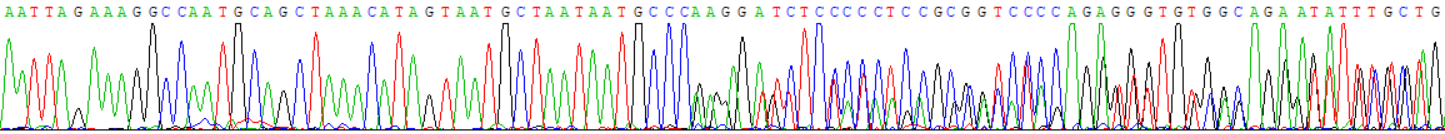
**

***CYP92B14*-sgRNA-L16**

**Allele1:   TAATGCTAATAATGCCCAAGTCGGAGTTCTCCATCAGCGGCATCCAGAGGTTGTG (insertion)**

**Allele2:   TAATGCTAATAATGCCCAAGTCGGAGTTCTCCATCAGCGGCATCCAGAGGTTGTG (insertion)**

**
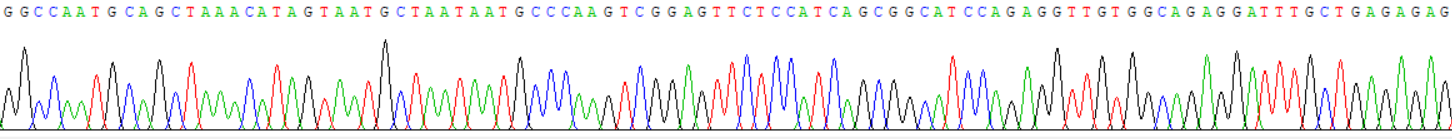
Reference: TAATGCTAATAATGCCCAAG-CGGAGTTCTCCATCAGCGGCATCCAGAGGTTGTG**

***CYP92B14*-sgRNA-L17**

**Allele1:   TAATGCTAATAATG****CCCAAGTCGGAGTTCTCCATCAGCGGCATCCAGAGGTTGTG (insertion)**

**Allele2:   TAATGCTAATAATGCCCAAGTCGGAGTTCTCCATCAGCGGCATCCAGAGGTTGTG (insertion)**

**Reference: TAATGCTAATAATGCCCAAG-CGGAGTTCTCCATCAGCGGCATCCAGAGGTTGTG**

**
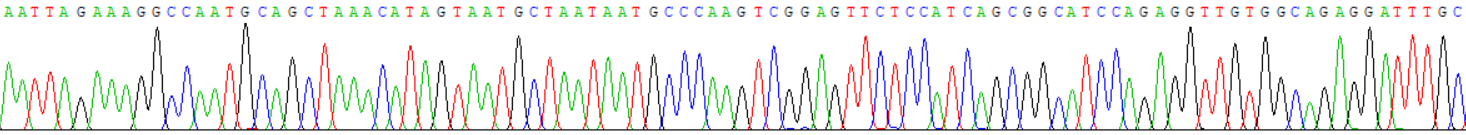
**

***CYP92B14*-sgRNA-L18**

**Allele1:   TAATGCTAATAATGCCCAAGACGGAGTTCTCCATCAGCGGCATCCAGAGGTTGT (insertion)**

**Allele2:   TAATGCTAATAATGCCCAAGACGGAGTTCTCCATCAGCGGCATCCAGAGGTTGT (insertion)**

**Reference: TAATGCTAATAATGCCCAAG-CGGAGTTCTCCATCAGCGGCATCCAGAGGTTGT**

**
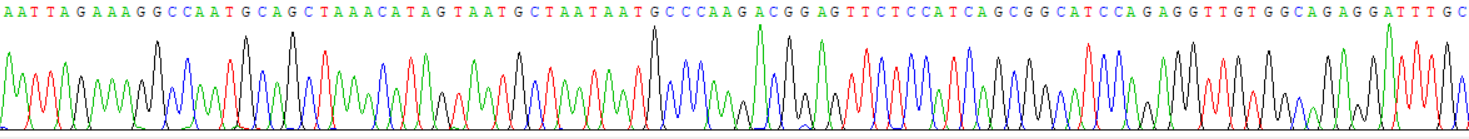
**

***CYP92B14*-sgRNA-L19**

**Allele1:   TGCTAATAATGCCCAAGCGGAGTTCTCCATCAGCGGCATCCAGAGGTTGTGGCAGAG (WT)**

**Allele2:   TGCTAATAATGCCCAAGCGGAGTTCTCCATCAGCGGCATCCAGAGGTTGTGGCAGAG (WT)**

**Reference: TGCTAATAATGCCCAAGCGGAGTTCTCCATCAGCGGCATCCAGAGGTTGTGGCAGAG**

**
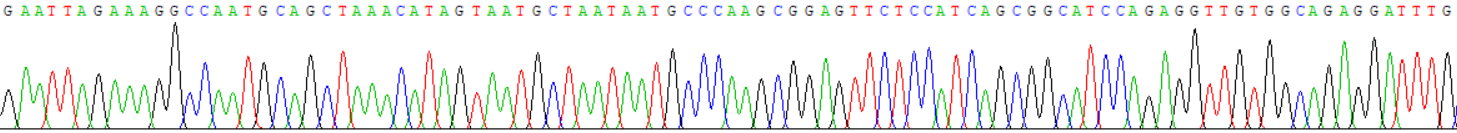
**

***CYP92B14*-sgRNA-L20**

**Allele1:   TAATGCTAATAATGCCCAAGTCGGAGTTCTCCATCAGCGGCATCCAGAGGTTGTGG (insertion)**

**Allele2:   TAATGCTAATAATGCCCAAGTCGGAGTTCTCCATCAGCGGCATCCAGAGGTTGTGG (insertion)**

**Reference: TAATGCTAATAATGCCCAAG-CGGAGTTCTCCATCAGCGGCATCCAGAGGTTGTGG**

**
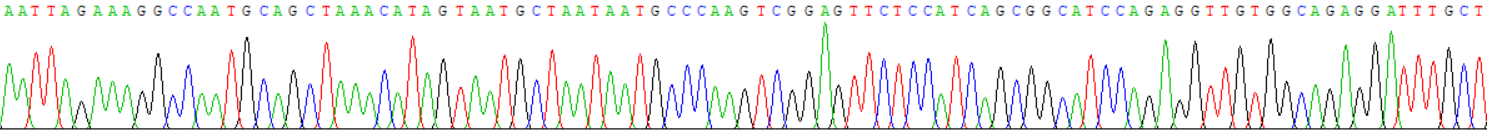
**

***CYP92B14*-sgRNA-L21**

**Allele1:   TAATGCTAATAATGCCCAAGGTCGGAGTTCTCCATCAGCGGCATCCAGAGGTTGTG (insertion)**

**Allele2:   TAATGCTAATAATGCCCAAGGTCGGAGTTCTCCATCAGCGGCATCCAGAGGTTGTG (insertion)**

**Reference: TAATGCTAATAATGCCCAAG--CGGAGTTCTCCATCAGCGGCATCCAGAGGTTGTG**

**
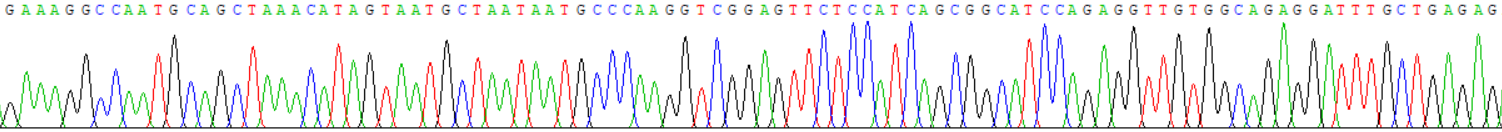
**

***CYP92B14*-sgRNA-L22**

**Allele1:   TAATGCCCAAG---------TCCATCAGCGGCATCCAGAGGTTGTGGCAGAGGATT (deletion)**

**Allele2:   TAATGCCCAAGTCGGAGTTCTCCATCAGCGGCATCCAGAGGTTGTGGCAGAGGATT (insertion)**

**Reference: TAATGCCCAAG-CGGAGTTCTCCATCAGCGGCATCCAGAGGTTGTGGCAGAGGATT**

**
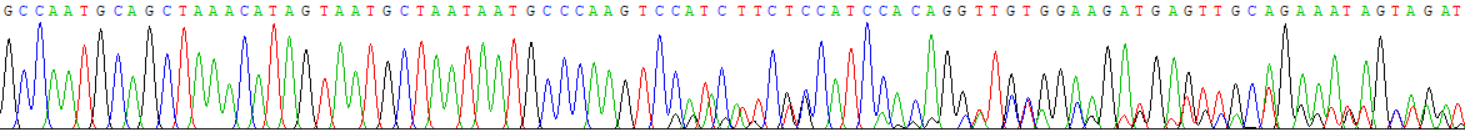
**

***CYP92B14*-sgRNA-L23**

**Allele1:   TAATGCTAATAATGCCCAAGTCGGAGTTCTCCATCAGCGGCATCCAGAGGTTGTG (insertion)**

**Allele2:   TAATGCTAATAATGCCCAAGTCGGAGTTCTCCATCAGCGGCATCCAGAGGTTGTG (insertion)**

**Reference: TAATGCTAATAATGCCCAAG-CGGAGTTCTCCATCAGCGGCATCCAGAGGTTGTG**

**
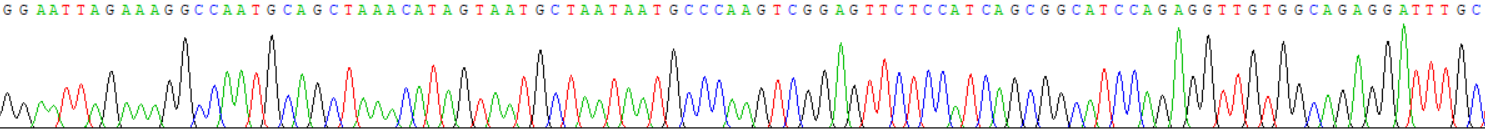
**

***CYP92B14*-sgRNA-L24**

**Allele1:   TAATGCTAATAATGCCCAAG----AGTTCTCCATCAGCGGCATCCAGAGGTTGTGGC (deletion)**

**Allele2:   TAATGCTAATAATGCCCAAGTCGGAGTTCTCCATCAGCGGCATCCAGAGGTTGTGGC (insertion)**

**Allele3:   TAATGCTAATAATGCCCAAGACGGAGTTCTCCATCAGCGGCATCCAGAGGTTGTGGC (insertion)**

**Reference: TAATGCTAATAATGCCCAAG-CGGAGTTCTCCATCAGCGGCATCCAGAGGTTGTGGC**

**
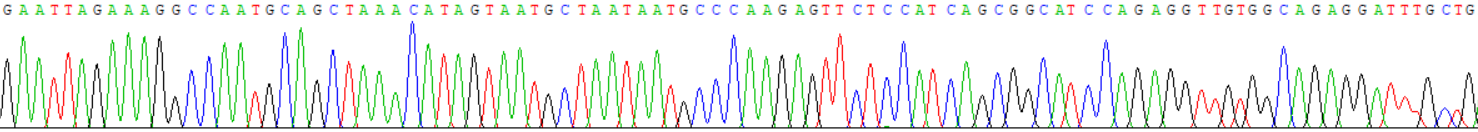
**

**
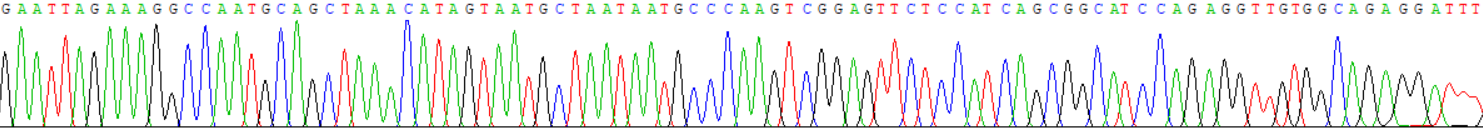
**

**
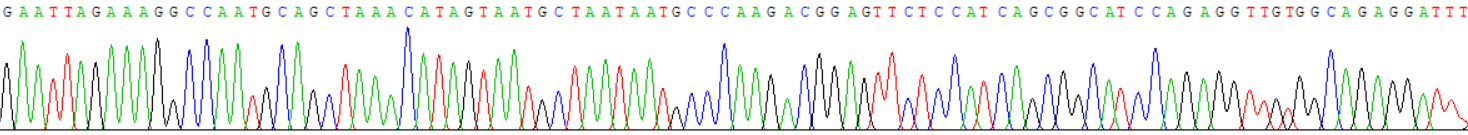
**

***CYP92B14*-sgRNA-L25**

**Allele1:   TAATGCTAATAATGCCCAAGACGGAGTTCTCCATCAGCGGCATCCAGAGGTTGTG (insertion)**

**Allele2:   TAATGCTAATAATGCCCAAGTCGGAGTTCTCCATCAGCGGCATCCAGAGGTTGTG (insertion)**

**Reference: TAATGCTAATAATGCCCAAG-CGGAGTTCTCCATCAGCGGCATCCAGAGGTTGTG**

**
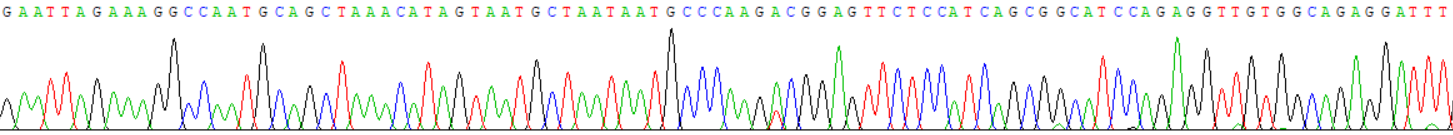
**

***CYP92B14*-sgRNA-L26**

**Allele1:   TGCTAATAATGCCCAAG-CGGAGTTCTCCATCAGCGGCATCCAGAGGTTGTGGCAGA (WT)**

**Allele2:   TGCTAATAATGCCCAAGGCGGAGTTCTCCATCAGCGGCATCCAGAGGTTGTGGCAGA (insertion)**

**Reference: TGCTAATAATGCCCAAG-CGGAGTTCTCCATCAGCGGCATCCAGAGGTTGTGGCAGA**

**
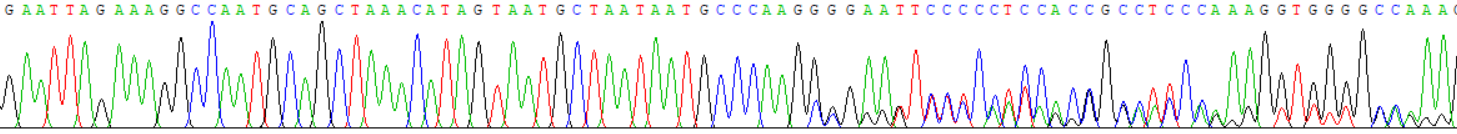
**

***CYP92B14*-sgRNA-L27**

**Allele1:   TAATGCTAATAATGCCCAAG-----------------------------GTTGTGGCAGAGGAT (deletion)**

**Allele2:   TAATGCTAATAATGCCCAAGTCGGAGTTCTCCATCAGCGGCATCCAGAGGTTGTGGCAGAGGAT (insertion)**

**Reference: TAATGCTAATAATGCCCAAG-CGGAGTTCTCCATCAGCGGCATCCAGAGGTTGTGGCAGAGGAT**

**
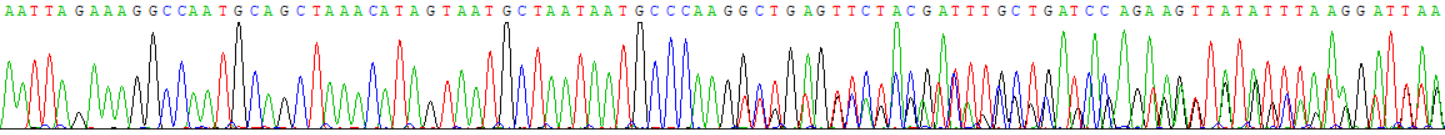
**

***CYP92B14*-sgRNA-L28**

**Allele1:   AATAATGCCCAAGCGGAGTTCTCCATCAGCGGCATCCAGAGGTTGTGGCAGAGGATTTGCTG (WT)**

**Allele2:   AATAATGCCCAAG--------TCCATCAGCGGCATCCAGAGGTTGTGGCAGAGGATTTGCTG (deletion)**

**Reference: AATAATGCCCAAGCGGAGTTCTCCATCAGCGGCATCCAGAGGTTGTGGCAGAGGATTTGCTG**

***
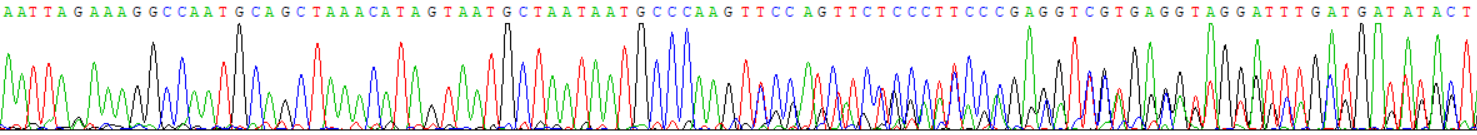
***

***CYP92B14*-sgRNA-L29**

**Allele1:   TAATGCTAATAATGCCCAAG---GGTTCTCCATCAGCGGCATCCAGAGG (deletion and insertion)**

**Allele2:   TAATGCTAATAATGCCCAAG---GGTTCTCCATCAGCGGCATCCAGAGG (deletion and insertion)**

**Reference: TAATGCTAATAATGCCCAAGCGGAGTTCTCCATCAGCGGCATCCAGAGG**


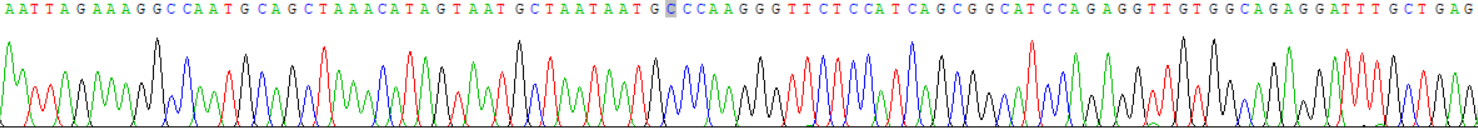


***CYP92B14*-sgRNA-L30**

**Allele1:   TAATGCTAATAATGCCCAAGTCGGAGTTCTCCATCAGCGGCATCCAGAGGTTGTGGCAGAGGA (insertion)**

**Allele2:   TAATGCTAATAATGCCCAAG----TGTTCTCCATCAGCGGCATCCAGAGGTTGTGGCAGAGGA (deletion)**

**Reference: TAATGCTAATAATGCCCAAG-CGGAGTTCTCCATCAGCGGCATCCAGAGGTTGTGGCAGAGGA**


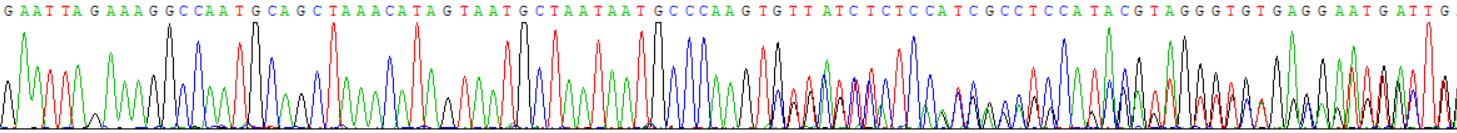

Supplement: Supplementary file 4 [file Data_Sheet_4.docx]
